# A harmonized meta-knowledgebase of clinical interpretations of somatic genomic variants in cancer

**DOI:** 10.1038/s41588-020-0603-8

**Published:** 2020-04-03

**Authors:** Alex H. Wagner, Brian Walsh, Georgia Mayfield, David Tamborero, Dmitriy Sonkin, Kilannin Krysiak, Jordi Deu-Pons, Ryan P. Duren, Jianjiong Gao, Julie McMurry, Sara Patterson, Catherine del Vecchio Fitz, Beth A. Pitel, Ozman U. Sezerman, Kyle Ellrott, Jeremy L. Warner, Damian T. Rieke, Tero Aittokallio, Ethan Cerami, Deborah I. Ritter, Lynn M. Schriml, Robert R. Freimuth, Melissa Haendel, Gordana Raca, Subha Madhavan, Michael Baudis, Jacques S. Beckmann, Rodrigo Dienstmann, Debyani Chakravarty, Xuan Shirley Li, Susan Mockus, Olivier Elemento, Nikolaus Schultz, Nuria Lopez-Bigas, Mark Lawler, Jeremy Goecks, Malachi Griffith, Obi L. Griffith, Adam A. Margolin, Variant Interpretation for Cancer Consortium

**Affiliations:** 1 Washington University School of Medicine, St. Louis, MO USA; 2 Oregon Health and Science University, Portland, OR USA; 3 Pompeu Fabra University, Barcelona, Spain; 4 Karolinska Institute, Solna, Sweden; 5 National Cancer Institute, Rockville, MD USA; 6 Institute for Research in Biomedicine, Barcelona, Spain; 7 Catalan Institution for Research and Advanced Studies, Barcelona, Spain; 8 MolecularMatch, Houston, TX USA; 9 Memorial Sloan Kettering Cancer Center, New York, NY USA; 10 The Jackson Laboratory for Genomic Medicine, Farmington, CT USA; 11 Dana–Farber Cancer Institute, Boston, MA USA; 12 Mayo Clinic, Rochester, MN USA; 13 Acibadem University, Istanbul, Turkey; 14 Vanderbilt University, Nashville, TN USA; 15 Charité—Berlin University of Medicine, Berlin, Germany; 16 Institute for Molecular Medicine Finland, Helsinki, Finland; 17 University of Turku, Turku, Finland; 18 Baylor College of Medicine, Houston, TX USA; 19 Texas Children’s Hospital, Houston, TX USA; 20 University of Maryland School of Medicine, Baltimore, MD USA; 21 Linus Pauling Institute at Oregon State University, Corvallis, OR USA; 22 Children’s Hospital Los Angeles, Los Angeles, CA USA; 23 Keck School of Medicine of USC, Los Angeles, CA USA; 24 Georgetown University Medical Center, Washington, DC USA; 25 University of Zurich, Zurich, Switzerland; 26 University of Lausanne, Lausanne, Switzerland; 27 Vall d’Hebron Institute of Oncology, Barcelona, Spain; 28 Weill Cornell Medicine, New York, NY USA; 29 Queen’s University Belfast, Belfast, UK

**Keywords:** Genetics research, Cancer

## Abstract

Precision oncology relies on accurate discovery and interpretation of genomic variants, enabling individualized diagnosis, prognosis and therapy selection. We found that six prominent somatic cancer variant knowledgebases were highly disparate in content, structure and supporting primary literature, impeding consensus when evaluating variants and their relevance in a clinical setting. We developed a framework for harmonizing variant interpretations to produce a meta-knowledgebase of 12,856 aggregate interpretations. We demonstrated large gains in overlap between resources across variants, diseases and drugs as a result of this harmonization. We subsequently demonstrated improved matching between a patient cohort and harmonized interpretations of potential clinical significance, observing an increase from an average of 33% per individual knowledgebase to 57% in aggregate. Our analyses illuminate the need for open, interoperable sharing of variant interpretation data. We also provide a freely available web interface (search.cancervariants.org) for exploring the harmonized interpretations from these six knowledgebases.

## Abstract (web summary)

This analysis presents a harmonized meta-knowledgebase to facilitate clinical interpretation of somatic genomic variants in cancer. This community-based project highlights the need for cooperative efforts to curate clinical interpretations of somatic variants for robust practice of precision oncology.

## Main

Precision oncology—in which treatment is informed by the mutational profile of a cancer—requires concise, standardized and searchable clinical interpretations of detected variants. Interpretations of biomarker–disease associations can be diagnostic, prognostic, therapeutic (predictive of favorable or adverse response to therapy) and/or predisposing (germline variants that increase risk of developing cancer). Many have curated the biomedical literature to collect and formalize these interpretations into knowledgebases^1–12^. These isolated efforts have resulted in disparate knowledge representation, and exchange of these biomarker–disease associations remains a difficult challenge^13^. Consequently, stakeholders interested in the effects of somatic cancer variants are faced with the following trade-off: (1) reconciling multiple representations and interpretations across knowledgebases; or (2) potentially omitting clinically significant interpretations that are not universally captured. Manual aggregation of information across knowledgebases to interpret the variant profile for each patient is an unsustainable approach at scale. Moreover, the lack of an integrated resource has precluded the ability to easily assess the current state of precision treatment options. Published reports^14–17^ have relied on individual, often highly discordant knowledgebases. Interoperability and automated aggregation are required to make a comprehensive approach to cancer precision medicine tractable and to establish consensus across knowledgebases.

The current diversity and number of ‘knowledge silos’ and the associated difficulties of coordinating these disparate knowledgebases have led to an international effort to maximize genomic data sharing^18,19^. The Global Alliance for Genomics and Health (GA4GH) has emerged as an international cooperative project to accelerate the development of approaches for responsible, voluntary and secure sharing of genomic and clinical data^20,21^. The Variant Interpretation for Cancer Consortium (VICC; cancervariants.org) is a Driver Project of GA4GH, established to co-develop standards for genomic data sharing (https://www.ga4gh.org/how-we-work/driver-projects/ga4gh.org/howwework/driver-projects.html). Specifically, the VICC is a consortium of clinical variant interpretation experts addressing the challenges of representing and sharing curated interpretations across the cancer research community.

Somatic variants in cancer-relevant genes are evaluated from multiple partially overlapping perspectives (Supplementary Note). The Association for Molecular Pathology, the American Society of Clinical Oncology and the College of American Pathologists (AMP/ASCO/CAP) have published structured somatic variant clinical interpretation guidelines that specifically address diagnostic, prognostic and therapeutic implications^22^. These guidelines do not provide systematic and comprehensive procedures to classify somatic variant oncogenicity, as has been published in the American College of Medical Genetics and Genomics (ACMG)/AMP guidelines^23^ for pathogenicity interpretation of germline variants.

Another common difference between somatic and germline classification is the frequent use of variant representations that are defined by multiple alternative genomic alterations, including protein variants such as NP_004295.2:p.F1174L (ALK F1174L; caused by either NC_000002.11:g.29443695G>T or NC_000002.11:g.29443695G>C), and categorical variants^24^, such as ‘loss-of-function mutations’ or ‘activating mutations’ (the use of the word ‘mutations’ in these variant names is a somatic-specific nomenclature that is common across these knowledgebases). This represents an important distinction from the interpretation of germline variants, which are typically described by singular and specific DNA variants, and only rarely in broader terms. A primary challenge of this work was to handle the complexity of these somatic variant representations.

We leveraged the VICC member expertise to aggregate cancer variant interpretations from six distinguished constituent knowledgebases: the Cancer Genome Interpreter Cancer Biomarkers Database (CGI), Clinical Interpretation of Variants in Cancer (CIViC), Jackson Laboratory Clinical Knowledgebase (JAX-CKB), MolecularMatch (MMatch), OncoKB and the Precision Medicine Knowledgebase (PMKB) (Supplementary Table 1)^1,5,9–11^. From a larger survey of published and available knowledgebases of clinical interpretations of genomic variants (Supplementary Table 1), these knowledgebases were selected for their similarity in somatic disease focus. The institutions leading each constituent knowledgebase agreed upon a core set of principles describing minimal data licensing and structure requirements (http://cancervariants.org/principles/ and Supplementary Note).

Our cooperative effort developed a framework for structuring and harmonizing clinical interpretations across these knowledgebases. Specifically, we defined key elements of variant interpretations (genes, variants, diseases, drugs and evidence), developed strategies for harmonization and implemented this framework to consolidate interpretations into a single, harmonized meta-knowledgebase (freely available at search.cancervariants.org).

## Results

### Aggregating and structuring interpretation knowledge

**Fig. 1:**
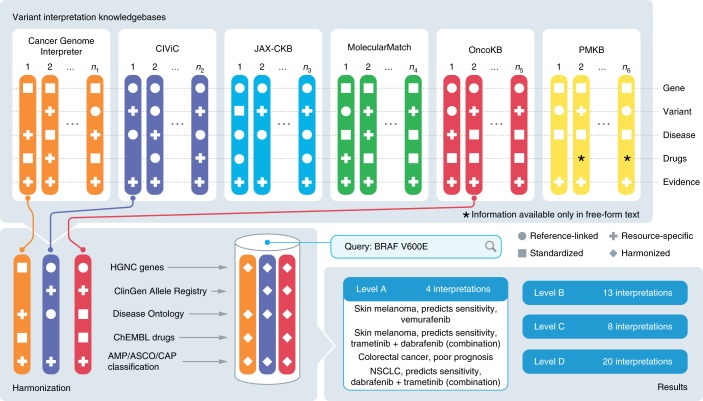
Creation of a harmonized meta-knowledgebase. Six variant interpretation knowledgebases of the VICC (top panel) and representative symbolic interpretations from each (colored columns) are illustrated. Interpretations are split across five different elements: gene, variant, disease, drugs and evidence, and are colored to indicate their originating knowledgebase. Reference-linked elements correspond to unique identifiers from established authorities for that element (for example, the use of Entrez or Ensembl gene identifiers). Standardized elements correspond to immediately recognizable formats or descriptions of elements, but are not linked to an authoritative definition. Resource-specific elements are described by terminology unique to the knowledgebase. These elements are each harmonized (bottom left panel) to a common reference standard (shown here is the use of HGNC for genes, ChEMBL for drugs, AMP/ASCO/CAP guidelines for evidence, Disease Ontology for diseases and ClinGen Allele Registry for variants). This harmonized meta-knowledgebase allows for querying across interpretations from each of the constituent VICC knowledgebases (bottom right panel, example query BRAF V600E), returning aggregated results, which are categorized and sorted by evidence level.

A review of the constituent somatic knowledgebases of the VICC (Fig. 1 and Supplementary Table 1)^1,5,9–11^ showed dramatic differences in the components of variant interpretations, which were often a mixture of concepts with standardized (such as Human Gene Nomenclature Committee (HGNC) gene symbols^25^, Human Genome Variation Society (HGVS) variant nomenclature^26^), externally referenced (identified elements of an established ontology or database) or knowledgebase-specific (shorthand, internal identifier) representations (Fig. 1). Representations of an element could vary within a knowledgebase, such as with the use of shorthand for diseases, including both standardized representations (for example, ‘CLL’ and ‘ALL’ are both listed synonyms in the NCI Thesaurus^27^) and internal representations (for example, ‘G’ (glioma), ‘L’ (lung cancer) or ‘OV’ (ovarian cancer)).

**Extended Data Fig. 1:**
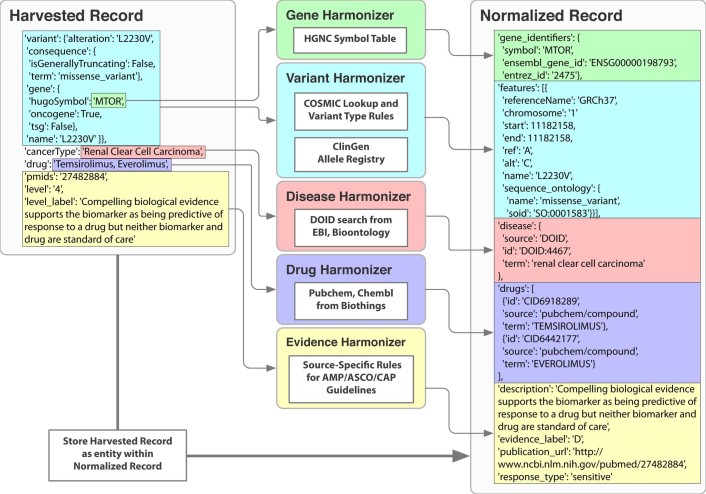
Harvesting and harmonizing records. Harvested interpretation records (left column) from each knowledgebase vary in structure, a consequence of how they are represented and exported by their parent knowledgebase. Knowledgebase-specific rules are written to select data from harvested records for harmonization across a suite of element-specific harmonizers (center column). Colors represent different elements of an interpretation, which are each harmonized independently: genes (green), variants (cyan), diseases (red), drugs (purple), and evidence (yellow). Outputs from these harmonizers are assembled into normalized records (right column).

We harmonized variant interpretations from each of these knowledgebases by mapping all data elements in each knowledgebase to established standards and ontologies describing genes, variants, diseases and drugs (Fig. 1 and Supplementary Note). Briefly, genes were harmonized using the HGNC gene symbols. Variants were harmonized through a combination of knowledgebase-specific rules, matching to the Catalog of Somatic Mutations in Cancer (COSMIC)^3^, and use of the ClinGen Allele Registry (reg.clinicalgenome.org)^28^. Diseases were harmonized using the European Bioinformatics Institute (EBI) Ontology Lookup Service (OLS; www.ebi.ac.uk/ols/index) to retrieve Disease Ontology (DO) terms and identifiers. Drugs were harmonized through queries to the Mychem.info API (mychem.info), PubChem^29^ and ChEMBL^30^. Details for each of these harmonization strategies are described in Methods and Extended Data Fig. 1.

Due to the knowledgebase-specific nature of describing an interpretation evidence level (Fig. 1), harmonization required manual mapping of evidence levels to a common standard. The AMP/ASCO/CAP somatic classification guidelines were released after (and partially informed by) the design of the VICC knowledgebases. These guidelines are compatible with (but not identical to) the existing evidence levels of these knowledgebases. We constructed a mapping of evidence levels provided by each knowledgebase to the evidence levels constituting AMP/ASCO/CAP tier I and II variants (Table 1).

**Table 1:** Mapping knowledgebase-specific evidence codes to AMP/ASCO/CAP guidelines

Evidence level	Defining characteristics	CIViC	OncoKB	JAX-CKB	CGI	MMatch	PMKB
Level A (tier I)	Evidence from professional guidelines or FDA-approved therapies relating to a biomarker and disease.	Level A	Level 1/2A /R1	Guideline/FDA approved	Clinical practice	Level 1A	Tier 1
Level B (tier I)	Evidence from clinical trials or other well-powered studies in clinical populations, with expert consensus.	Level B	Level 3A	Phase III	Clinical trials III–IV	Level 1B	
Level C (tier II)	Evidence for therapeutic predictive markers from case studies, or other biomarkers from several small studies. Also, evidence for biomarker therapeutic predictions for established drugs for different indications.	Predictive level C	Level 2B, level 3B	Clinical study/phase I/phase II	Clinical trials I–II, case reports	Level 2C	Tier 2
Level D (tier II)	Preclinical findings or case studies of prognostic or diagnostic biomarkers. Also includes indirect findings.	Nonpredictive level C/level D/level E	Level 4	Phase 0, preclinical	Preclinical data	Level 2D	

### The landscape of variant interpretation knowledge

**Extended Data Fig. 2:**
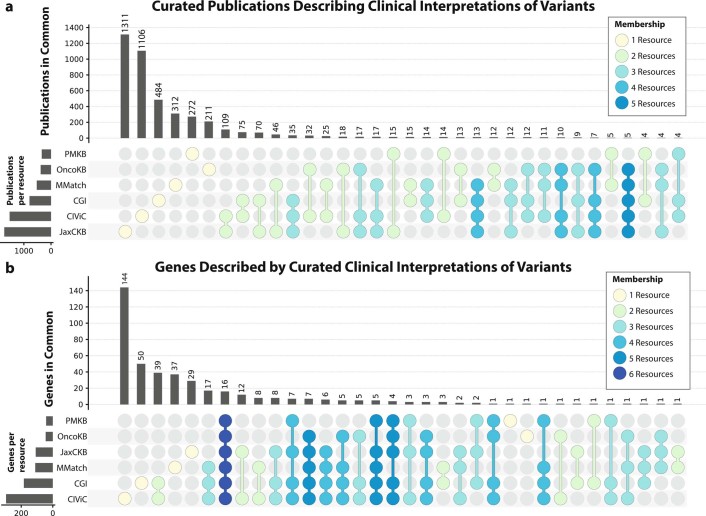
Knowledgebase overlap. **a**, Upset plot of publications supporting clinical interpretations of variants. The overwhelming majority of publications are observed in only 1 of 6 resources. **b**, Upset plot of genes described by clinical interpretations of variants. Compared to other interpretation elements, genes are much more commonly shared between resources.

The meta-knowledgebase v.0.10 release contained 12,856 harmonized interpretations (hereafter referred to as the core dataset; Methods) supported by 4,354 unique publications for an average of 2.95 interpretations per publication. Notably, 83% of all publications were referenced by only one knowledgebase, and only one publication^31^ was referenced across all six knowledgebases (Extended Data Fig. 2a). Gene symbols were almost universally provided; the few interpretations lacking gene symbols (<0.01%) were structural variants that were not associated with an individual gene. In contrast to publications, the genes curated by the cancer variant interpretation community are much more frequently observed in multiple knowledgebases. We observed that 23% of genes (97/415) with at least one interpretation were present in at least half of the knowledgebases, compared to only 5% of publications (203/4,354; odds ratio, OR = 1.6 × 10^−1^, *P* = 4.7 × 10^−34^; Fisher’s exact test, two-sided; Extended Data Fig. 2b).

**Fig. 2:**
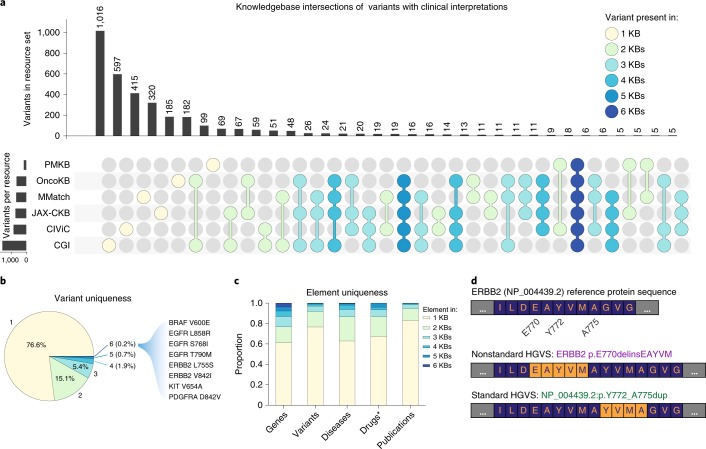
Representation of genomic variants across interpretation knowledgebases. **a**, UpSet plot^46^ of variants across six cancer variant interpretation knowledgebases (KBs). Sets of variant interpretation knowledgebases with shared variants are indicated by colored dots in the lower panel, with color indicating set size (for example, yellow dots indicate only the single designated knowledgebase in the set, green dots indicate two knowledgebases in the set, etc.). Objects are attributed to the largest containing set; thus, a variant described by all six knowledgebases is attributed to the dark blue set with eight variants. **b**, Pie chart visualizing overall uniqueness of variants, with categories indicating the number of knowledgebases describing each variant. Nearly 77% of variants are unique across the knowledgebases, with only 0.2% ubiquitously represented. The eight variants present in all six knowledgebases are listed on the right. **c**, A comparison of element uniqueness across knowledgebases. Despite having the greatest degree of overlap across all elements, approximately 61% of genes are unique across the knowledgebases. Literature cited to support interpretations has the smallest degree of overlap across all elements, with 83% of publications remaining unique across the knowledgebases. *Drugs are not evaluated for PMKB, which does not formally represent this concept. **d**, Multiple syntactically valid representations of an identical protein product can lead to confusion in describing the change in the literature and in variant databases. The wild-type protein sequence (dark blue with orange lettering) is represented for ERBB2 (top). Two (of many) possible representations of an inframe insertion (orange with dark blue lettering) are shown (bottom). A nonstandard HGVS expression describes a five-amino-acid insertion replacing one glutamate residue (middle). At the bottom, the HGVS standard representation shows an identical protein product from a four-amino-acid duplication. A search for one representation against a database with another (nonoverlapping) representation may lead to omission of a clinically relevant finding.

Variants had little overlap across the core dataset (Fig. 2a). Of the constituent 3,439 unique variants, 76.6% were described by only one knowledgebase, and <10% were observed in at least three (Fig. 2b). This lack of overlap was partially due to the complexity of variant representation. For example, the representation of an ERBB2 variant as described in nomenclature defined by the HGVS^26^ is NP_004439.2:p.Y772_A775dup, and yet it is referenced in multiple different forms in the biomedical literature. p.E770delinsEAYVM^32^, p.M774insAYVM^33^ and p.A775_G776insYVMA^34^ all describe an identical protein kinase domain alteration, although they appear to identify different variants (Fig. 2c). Despite having a standard representation by the HGVS guidelines, these alternative forms continue to appear in the literature. Consequently, a researcher looking to identify a specific match to NP_004439.2:p.E770delinsEAYVM may find no direct matches, although several exist under various alternate representations. This component of variant harmonization was addressed through the use of the ClinGen Allele Registry (Methods). Some differences in the scale and structure of these knowledgebases may be attributed to curation strategies (Supplementary Note).

To illustrate the challenges of searching across multiple variant representations, we surveyed all interpretations describing the previously discussed ERBB2 variant (NP_004439.2:p.Y772_A775dup) using the web interfaces provided by each knowledgebase (Table 2 and Supplementary Table 2). Each knowledgebase represented this variant differently. Two did not have specific interpretations for this variant, although they did have relevant categorical variants (for example, ‘exon 20 insertions’; Table 2). Most of the knowledgebases had a single internal representation of the variant, although the majority of these representations did not match across knowledgebases. The evidence describing these interpretations varied considerably in form, as each used knowledgebase-specific nomenclature (for example, evidence described as ‘level 3A’ in OncoKB is equivalent to ‘level 1B’ from MolecularMatch, or ‘level B’ from CIViC; Tables 1 and 2). Of the 19 unique publications describing the collected evidence, only three were observed in more than one knowledgebase, and none were observed in more than two. Interestingly, the curated interpretations from these shared publications varied by knowledgebase in disease scope (‘advanced solid tumor’ compared to ‘non-small cell lung cancer’ (NSCLC)^35^; ‘breast cancer and NSCLC’ compared to ‘cancer’^36^). A review of the interpretations showed some that are present in most of the knowledgebases (for example, ‘use of afatinib, trastuzumab or neratinib in NSCLC’; Table 2), and others that are present in only one or two (for example, ‘use of lapatinib in lung adenocarcinoma’ and ‘use of afatinib and rapamycin in combination in NSCLC’; Table 2). Importantly, this includes sparse interpretations that describe conflicting evidence (for example, ‘no benefit from neratinib in NSCLC’; Table 2) or negative evidence (for example, ‘does not support sensitivity/response to dacomitinib in NSCLC’; Table 2). Collectively, these data illustrate the diversity in knowledgebase structure, content, terminology and curation methodology. Consequently, utilizing a subset of these knowledgebases would likely result in differing interpretations before the harmonization performed in this study.

**Table 2:** Comprehensive assessment of the NP_004439.2:p.Y772_A775dup variant across clinical interpretation knowledgebases

Resource	ERBB2 variant name	Evidence	Document ID	Interpretation
CIViC	M774INSAYVM	Level B, 2-star	PMID: 25899785	Does not support sensitivity/response to dacomitinib in NSCLC
	M774INSAYVM	Level C, 4-star	PMID: 26559459	Supports sensitivity/response to afatinib in lung adenocarcinoma
	M774INSAYVM	Level C, 3-star	PMID: 22325357	Supports sensitivity/response to afatinib in lung adenocarcinoma
	M774INSAYVM	Level C, 3-star	PMID: 25789838	Supports sensitivity/response to trastuzumab emtansine in lung adenocarcinoma
	M774INSAYVM	Level D, 3-star	PMID: 19122144	Supports sensitivity/response to afatinib and rapamycin (combination) in NSCLC
	Kinase domain mutation	Level C, 4-star	PMID: 26598547	Supports sensitivity/response to trastuzumab in lung adenocarcinoma
	Kinase domain mutation	Level C, 3-star	PMID: 22325357	Supports sensitivity/response to afatinib in lung adenocarcinoma
OncoKB	Exon 20 insertions	Level 4	10.1158/1538-7445.AM2016-2644	Supports response to AP32788 in NSCLC
	Oncogenic mutations	Level 3A	PMID: 23220880	Supports response to neratinib in breast cancer and NSCLC
			10.1158/1538-7445.AM2017-CT001	
CGI	inframe insertion A775YVMA	Early trials	10.1200/JCO.2017.35.15_suppl.8510	Responsive to ado-trastuzumab emtansine in lung cancer
	inframe insertion A775YVMA	Early trials	10.1158/1538-7445.AM2017-CT001	Responsive to neratinib in cancer
	proximal exon 20	Early trials	PMID: 26598547	Responsive to afatinib, neratinib, lapatinib or trastuzumab in lung adenocarcinoma
			10.1200/JCO.2017.35.15_suppl.9071	
PMKB	exon(s) 20 insertion	Tier 2	PMID: 22761469	Associated with sensitivity to some ERBB2 inhibitors in lung adenocarcinoma
			PMID: 16818618	
			PMID: 25152623	
JAX-CKB	Y772_A775dup	Clinical study	PMID: 26964772	Conflicting response to afatinib in lung adenocarcinoma
	Y772_A775dup	Phase II	PMID: 29420467	Predicted sensitive to neratinib in Her2-receptor-negative breast cancer
	Y772_A775dup	Phase II	PMID: 29420467	Predicted resistant to neratinib in urinary bladder cancer and NSCLC
	Y772_A775dup	Preclinical	PMID: 26545934	Sensitive to afatinib in lung cancer
	Y772_A775dup	Preclinical	PMID: 26545934	No benefit to gefitinib in lung cancer
	Y772_A775dup	Preclinical	PMID: 28363995	Sensitive to neratinib in advanced solid tumor
	exon 20 insertion	Clinical study	PMID: 28167203	Predicted sensitive to afatinib or trastuzumab in NSCLC
	exon 20 insertion	Clinical study	PMID: 26964772	Predicted sensitive to afatinib in lung adenocarcinoma
	exon 20 insertion	Phase II	PMID: 29420467	Predicted sensitive to neratinib in Her2-receptor-negative breast cancer
	exon 20 insertion	Phase II	PMID: 29420467	No benefit to neratinib in NSCLC
	exon 20 insertion	Preclinical	10.1158/1538-7445.AM2016-2644	Sensitive to AP32788 in advanced solid tumor
MolecularMatch	Y772_A775dup	Level 1B	PMID: 22325357, 26964772	Confers sensitivity to afatinib in patients with neoplasm of lung
	Y772_A775dup	Level 2C	PMID: 26598547	Confers sensitivity to trastuzumab in patients with neoplasm of lung
	Y772_A775dup	Level 2D	PMID: 22325357	Confers sensitivity to afatinib in patients with neoplasm of breast
	A775_G776insYVMA	Level 1A	PMID: 26559459, 22325357, 26545934	Confers sensitivity to afatinib in patients with neoplasm of lung
	A775_G776insYVMA	Level 2C	PMID: 23610105, 26964772, 22908275	Confers sensitivity to afatinib in patients with neoplasm of breast
	A775_G776insYVMA	Level 2D	PMID: 17311002, 22908275	Confers sensitivity to neratinib in patients with neoplasm of breast

### Harmonization improves consensus across interpretations

To test the effect of our harmonization methods on generating consensus, we evaluated the overlap of unique interpretation elements from each knowledgebase of the core dataset in comparison to unharmonized (but aggregated) data (Methods). As noted above, genes from each resource used HGNC gene symbols, resulting in very little gain from harmonization; 45% of genes across knowledgebases overlapped without harmonization, compared to 46% with harmonization. This is in contrast to variants (8% overlapping unharmonized, 26% overlapping harmonized), diseases (27% unharmonized, 34% harmonized) and drugs (20% unharmonized, 36% harmonized) (Supplementary Table 3). None of the evidence levels were consistent across resources when unharmonized, and all are consistent with a common standard (Table 1) after harmonization, which is a primary contribution of this work.

Notably, in some cases, harmonization dramatically increased the number of elements to be considered. For example, CGI had an increase in variant count from 283 (unharmonized) to 1,600 (harmonized) due to the expansion of ambiguous categorical variants (for example, ‘oncogenic mutation’) to the set of variants considered oncogenic by CGI (through extraction and mapping of the CGI Catalog of Validated Oncogenic Mutations). As mentioned above, the PMKB does not have a formalized ‘drug’ field for interpretations, so there is no reasonably accessible data for aggregating or harmonizing drugs for that resource. Drugs and variants both had a relatively greater benefit from normalization compared to the other interpretation elements, which was likely driven by the diverse and numerous synonymous representations of these concepts in use. While the complexities of variant representation have been discussed above, the complexity of drug labeling in these resources is driven by the multiple synonyms given to drugs in their numerous formulations and brands, which change relatively frequently over time.

### Harmonization increases findings of clinical significance

**Fig. 3:**
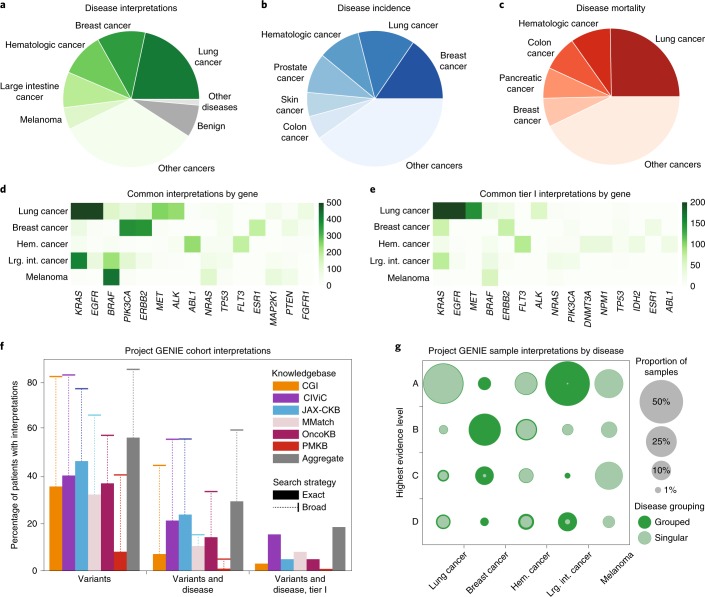
Clinical interpretations of variants are defined by disease. **a**–**c**, Core dataset interpretations for top-level disease groups. Distinct diseases are shown if the constituent interpretations for that disease account for at least 5% of the total dataset (**a**). Diseases accounting for at least 5% of cancer incidence (**b**) and mortality (**c**) are also displayed. Approximately 8% of interpretations are categorized as benign neoplasms (dark gray; for example von Hippel–Lindau disease). An additional 1% are categorized under high-level terms other than DOID:14566, disease of cellular proliferation. **d**,**e**, Heat map of frequent gene–disease interpretations (**d**) and the related heat map limited to tier 1 interpretations (**e**). **f**, Percentage of Project GENIE cohort with at least one interpretation from the indicated knowledgebase that matches patient variants (left group), patient variants and disease (center group) or patient variants, disease and a tier I evidence level (right group). A broader search strategy (indicated by whisker bars; Extended Data Fig. 4) that allows for regional variant matches (for example, gene level) and broader interpretation of disease terms (for example, DOID:162, cancer) nearly doubles the number of patients with matching interpretations. These broader match strategies are incompatible with the ASCO/AMP/CAP evidence guidelines. **g**, Most significant finding (by evidence level) across patient samples, by disease. Each column represents one of the common diseases indicated in **a**, and the rows represent the evidence levels described in Table 1. Inner, light green circles (labeled Singular) indicate the proportion observed when matching patient diseases to interpretations with the same disease ontology term. Outer, dark green circles (labeled Grouped) indicate the proportion observed when matching patients to interpretations with ancestor or descendant terms that group to the same class of disease (Methods). Hem. cancer, hematological cancer; Lrg. int. cancer, large intestine cancer.

**Extended Data Fig. 3:**
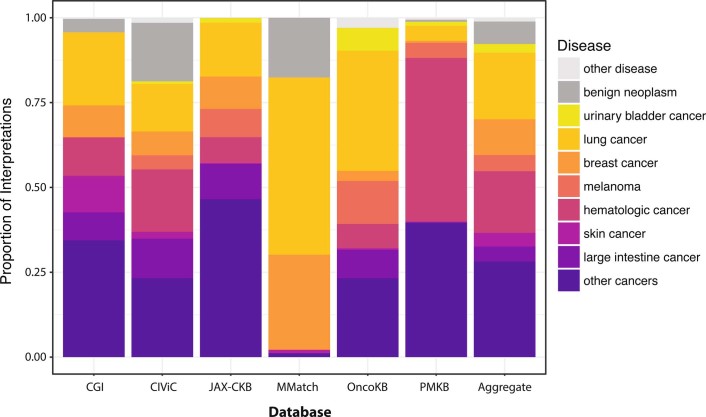
Knowledgebase disease enrichment. Relative distribution of interpretations describing diseases across the VICC resources. Several resources are strongly enriched for one or more diseases compared to the entire dataset (see related Supplementary Table 8).

Evaluation of patient variants for strong clinical significance requires an assessment of these variants in the appropriate disease context. When grouped to the nearest top-level disease term (Supplementary Table 4 and Supplementary Note), five major cancer group terms each accounted for over 5% of all interpretations in the core dataset: lung cancer (24%), breast cancer (13%), hematologic cancer (11%), large intestine cancer (9%) and melanoma (6%) (Fig. 3a and Supplementary Table 5). Notably, the most common interpretations mirror top-level terms that have both high incidence (Fig. 3b) and high mortality (Fig. 3c) as reported by the American Cancer Society (Supplementary Table 6)^37^: lung cancer, breast cancer and hematologic cancer. The ‘large intestine cancer’ term contains numerous interpretations describing colorectal cancers, which are closely related to colon cancer (a top-five cancer in both incidence and mortality; Supplementary Table 7). Evaluation of these terms across the core dataset showed significant differences in the distribution of common cancer types constituting each knowledgebase, illustrating the value of aggregating knowledgebases for a more comprehensive landscape of interpretations (Extended Data Fig. 3 and Supplementary Table 8).

**Extended Data Fig. 4:**
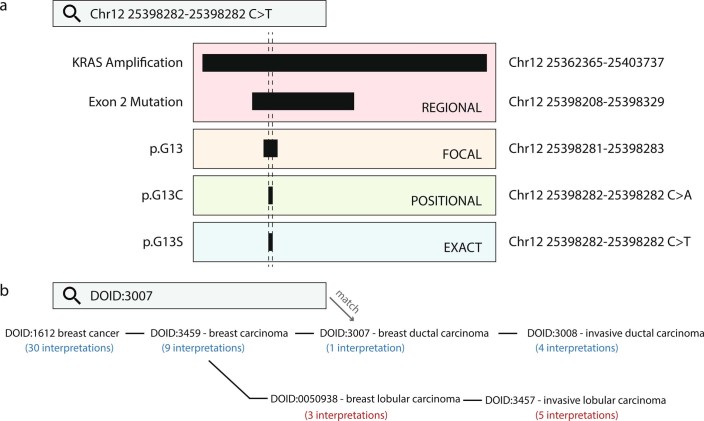
Search strategies. **a**, A variant intersection search strategy. Variants that match at position and allele are referred to as “exact” (blue box), variants matching at position only as “positional” (green box), variants that largely (but not completely) intersect are considered “focal” (orange box), and variants that overlap only a small amount are considered “regional” (red box). The left column shows matched results for a query (search box, top), based on the intersection of coordinates in the right column. **b**, TopNode disease search strategy. Shown are a subset of disease nodes that all map to the parent TopNode DOID:1612, ‘Breast Cancer’. A query for DOID:3007 would return 44 interpretations (blue) from the queried term, its direct ancestors (DOID:3459, ‘Breast Carcinoma’ and DOID:1612, ‘Breast Cancer’) and descendants (DOID:3008, ‘invasive ductal carcinoma’), but no interpretations (red) from indirectly related terms (DOID:0050938, ‘breast lobular carcinoma’ and DOID:3457, ‘invasive lobular carcinoma’).

**Extended Data Fig. 5:**
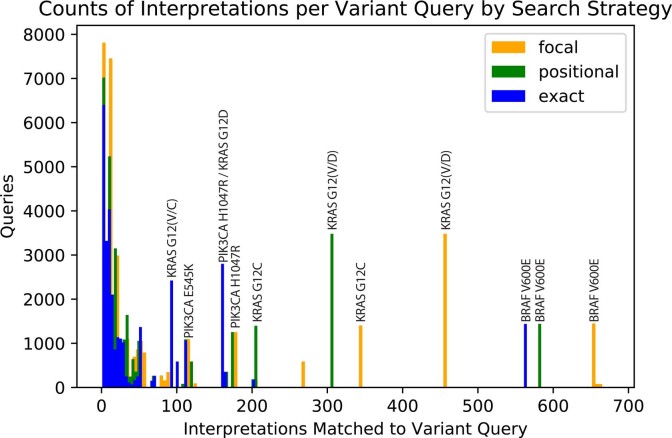
Commonality of observed mutations and their interpretations. Interpretation count (x-axis) by number of queries (y-axis). Focal (yellow) and positional (green) searches provide a benefit to interpretability over exact matching. Notably, several high interpretation spikes are observed, due to variants that have both a large number of interpretations and are often observed in the GENIE cohort. These include KRAS G12 mutations, BRAF V600E, and several mutations in PIK3CA.

To further test the value of harmonized interpretation knowledge, we evaluated the 38,207 patients of the AACR Project Genomics Evidence Neoplasia Information Exchange (GENIE)^38^. We first queried the 237,175 moderate- or high-impact variants from GENIE using a broad search strategy (Methods and Extended Data Fig. 4). Notably, 11% (4,355) of patients lacked any variants to search before filtering on predicted impact, and 12% (4,543) after. This search yielded 2,316,305 interpretation search results for an average of 9.8 interpretations per variant query. For a point mutation, these interpretations included matches to alternate alleles at the same position, associated amino acid changes, the exon or functional domain or gene-level interpretations such as overexpression, gain/loss-of-function or simply mutations. Restricting to a positional match (and thus excluding gene-level interpretations; Extended Data Fig. 4) showed an interpretation result set dominated by a few common GENIE point mutations in variants each with a large number of interpretations, including BRAF NP_004324.2:p.V600E, KRAS NP_004976.2:p.G12 mutations and both NP_006209.2:p.E545K and NP_006209.2:p.H1047R mutations in PIK3CA (Extended Data Fig. 5). This is congruent with our observation that the interpretations of the core dataset for the most common diseases are highly focused on these and other specific genes (Fig. 3d), including tier I interpretations (Fig. 3e). Examining our results at the patient level showed that a focused, variant-level search resulted in at least one interpretation (in any cancer type with any level of evidence) for 57% of patients in the GENIE cohort, compared to the average 33% obtained when using each constituent knowledgebase individually (Fig. 3f). We observed that broadening the search scope to include any regional match (Extended Data Fig. 4) increased the cohort coverage to 86% of patients (compared to an average of 68% per individual knowledgebase). However, it is prudent to keep in mind that the increase in matching percentage using regional match instead of exact match would be partly due to nononcogenic passenger variants.

**Extended Data Fig. 6:**
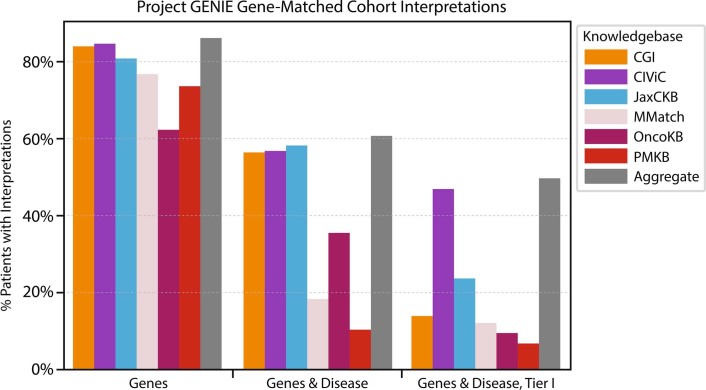
Gene intersection search. Percentage of Project GENIE cohort with at least one interpretation from the indicated knowledgebase that matches patient variant genes (left group), patient variant genes and disease (center group), or patient variant genes, disease, and a Tier I evidence level (right group). This very broad match strategy is incompatible with the ASCO/AMP/CAP evidence guidelines.

A key component in determining the clinical relevance of an interpretation is whether the tumor type reported in the interpretation matches the patient’s tumor type (see ‘Defining characteristics’ in Table 1). Restricting patient search results to those interpretations that are of matching grouped disease terms (Extended Data Fig. 4 and Supplementary Note) resulted in 29% of patients with at least one clinical interpretation (compared to an average individual knowledgebase match rate of 13%), and 18% of patients with at least one tier I clinical interpretation (compared to an average 6% per individual knowledgebase) (Fig. 3f). Patients with rare diseases were disadvantaged in this analysis, as automated mapping of their disease terms to DO was less likely to succeed (Supplementary Note). Allowing matching to any ancestor or descendant term and allowing partial variant overlaps improves matches to 60% (compared to an average of 35% per individual knowledgebase). This broader strategy, however, requires contextual re-evaluation of assigned AMP/ASCO/CAP evidence levels, which are designated for a precise match to variant and disease context. Consequently, evidence level or tier filtering can only be used with an exact search strategy. We evaluated an alternative, highly permissive search strategy that matches sample variants to any interpretation in the gene (Extended Data Fig. 6). The resulting match profile across the knowledgebases is comparable to findings from the overlapping variant strategy, indicating that many of the commonly mutated genes have gene-level interpretations (which would be a match by either strategy).

A comparison of interpretations across the previously described common cancers (with proportion >5% in Supplementary Table 5) showed that the use of grouped terms instead of exact terms for matching interpretations to patients’ cancers varies dramatically by cancer type, with some cancers (for example, lung cancer and melanoma) showing little increased interpretation breadth, while others have enormous effect (for example, breast cancer and large intestine cancer; Fig. 3g). This is primarily due to the specific nature by which patients are classified with certain diseases, versus the aggregate nature by which interpretations are ascribed to diseases. Interestingly, 56% of GENIE patient samples (6,196/11,149) have disease-matched interpretations across the frequently observed cancers, compared to only 40% (5,430/13,724) of patient samples across all other cancers (OR = 1.9, *P* = 3.9 × 10^−140^; Fisher’s exact test, two-sided). These numbers are reduced to 44% (4,881/11,149) and 18% (2,438/13,724), respectively, when considering only tier I interpretations (OR = 3.6, *P* < 2.2 × 10^−308^; Fisher’s exact test, two-sided).

### A resource for searching variant interpretation knowledge

**Fig. 4:**
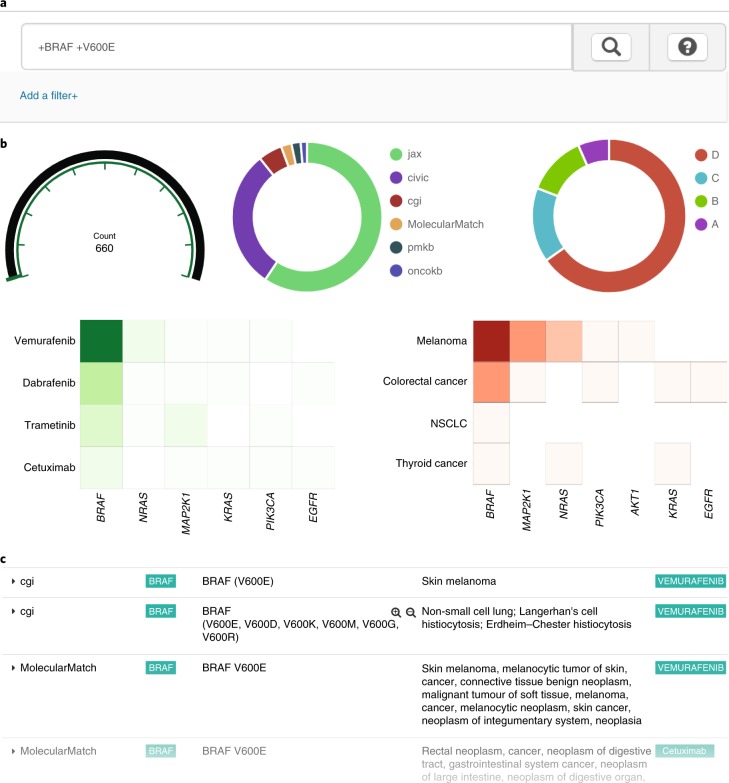
A web client for exploring the VICC meta-knowledgebase. **a**, Queries are entered as individual terms, with compound queries (for example, *BRAF* and V600E) denoted by preceding ‘+’ characters. Usage help and example documentation can be found by clicking the ‘?’ icon. **b**, Result visualization panels are interactive, allowing users to quickly filter results by evidence level, source, disease, drug and gene. **c**, Scrollable results table has sortable columns detailing each resource (for example, MolecularMatch), gene (*BRAF*), variant (V600E), disease (skin melanoma), drug (vemurafenib), evidence level, evidence direction, original URL and primary literature. Rows are expandable and include additional detail structure as both JavaScript object notation (JSON) and a table.

We have developed and hosted a public web interface for exploring the VICC meta-knowledgebase, freely available at search.cancervariants.org. This interface retrieves the most recent data release from an ElasticSearch index. Searching the knowledgebase is performed by specifying filters for any term or entering free text or compound (for example, and/or logic) queries in the search box at the top of the page (Fig. 4a). Panels with data distribution visualizations describe the current result set (Fig. 4b). These interactive panels provide additional information about specific subsets and may be used to create additional filters (for example, clicking on a level in the ‘evidence_level’ panel filters results throughout the page to display only those interpretations with the designated evidence level). This allows investigators to see the distribution of interpretations by evidence level, disease, gene and drug, and to filter according to their interests. Tabulated results are provided at the bottom of the page (Fig. 4c), and are expandable with all details, including the (unharmonized) record provided by the original knowledgebase for each interpretation. These search tools are available via both the web interface and an application programming interface (API) search endpoint (Methods), in addition to a GA4GH beacon on beacon-network.org. Additionally, a Python interface and analysis workbook have been developed to enable reproduction (and additional exploration) of the data presented in this paper, as well as full downloads of the underlying data (Methods). Usage documentation and example queries for this resource may be found at docs.cancervariants.org.

## Discussion

In this study, we aggregated, harmonized and analyzed clinical interpretations of cancer variants from six major knowledgebases^1,5,9–11^. Our analysis uncovered highly disparate content in curated knowledge, structure and primary literature across these knowledgebases. Specifically, we evaluated the unique nature of the vast majority of genomic variants reported across these knowledgebases and demonstrated the challenge of developing a consensus interpretation given these disparities. These challenges are exacerbated by nonstandard representations of clinical interpretations, in both the primary literature and curated knowledge of these resources. It is encouraging that the curators of these knowledgebases have, without coordination, independently curated diverse literature and knowledge sources. However, this reflects an enormous curation burden generated from the increasingly employed molecular characterizations of patient tumors and the related expansion of the primary literature describing them. Even at the gene level, for which there is the highest degree of overlap across any element of an interpretation, 61% of genes with interpretations are observed in only one knowledgebase. Our findings thus highlight the need for a cooperative, global effort to curate comprehensive and thorough clinical interpretations of somatic variants for robust practice of precision medicine.

We observed that harmonization improved concordance between interpretation elements across resources (Supplementary Note), and as a result we were able to achieve at least one specific (position-matched) harmonized variant interpretation for 57% of the patients in the GENIE cohort. In the most stringent searches, we required a precise variant match to a tier I interpretation also matching the patient’s cancer; in these cases, 18% of the cohort had a finding of strong clinical significance. Notably, these findings were substantially higher in patients with more common cancers, with 39% of the common cancer samples variant matching at least one tier I interpretation, compared to 15% of other cancer samples. These findings are concordant with observations of matched therapy rates in precision oncology trials, including 15% from IMPACT/COMPACT^15^, 11% from MSK-IMPACT^14^, 5% from the MD Anderson Precision Medicine Study^16^ and 23% from the NCI-MATCH trials^17^.

Collectively, our results portray a confluence of knowledge describing the most common genomic events relevant to the most frequent cancers, with highly disparate knowledge describing less frequent events in rare cancer types. The differing content of these knowledgebases may be a result of research programs targeted at frequent cancers, highlighting a need for a broader focus on less common cancers. This sparse landscape of curated interpretation knowledge is exacerbated by paucity in cross-references between ontologies describing disease, highlighting the importance of bridging this gap^39^. Similarly, complexities in variant representation have elucidated a need for sophisticated methods to harmonize genomic variants; harmonization with the ClinGen Allele Registry^28^ is suited to point mutations and indels, but the representation and harmonization of complex and nongenomic (for example, expression or epigenetic) variants remains a challenge.

Our harmonized clinical interpretation meta-knowledgebase represents a significant step forward in building consensus that was previously unattainable due to a lack of harmonization services, such as the Allele Registry, and expert standards and guidelines, such as those recommended by AMP/ASCO/CAP. This meta-knowledgebase serves as an open resource for evaluating interpretations from institutions with distinct curation structure, procedures and objectives. Potential uses include expert-guided therapy matching, supporting FDA regulatory processes associated with laboratory-developed genomic tests for guiding therapy and identification of diseases and biomarkers that warrant future study. The meta-knowledgebase web application is available at search.cancervariants.org, with usage documentation and examples at docs.cancervariants.org. The content of this meta-knowledgebase is dynamic, as we routinely poll the constituent knowledgebases for their associations between variants and clinical interpretations, which primarily comprise predictions of somatic variant effect on disease response to a therapy. Unlike the recently FDA-recognized ClinGen Expert Curated Human Variant Data^40,41^, this resource is not meant to be used to directly annotate clinical reports, but rather to serve as a search tool for existing knowledge pertaining to observed genomic variation.

While our initial efforts provide a structure by which variant interpretation knowledgebases can contribute to a broader and more consistent set of interpretations, much work remains to be done. In particular, VICC members contribute to GA4GH Work Streams to develop and integrate new and existing^42–45^ standards for the representation of variant interpretations and the evidence that describe them. Our web interface is being redesigned to a full-scale web service and user interface to concisely represent the most relevant interpretations for one or more variants. Specifically, we plan to add visual elements depicting the distribution of diseases corresponding to a searched variant, search modes specific to user intent (for example, disease-focused search, gene-focused search or multivariant search) and restyled result summaries. These and other planned changes are tracked on our central repository at git.io/metakb (Supplementary Note for other planned improvements).

In conclusion, there is a great need for a collaborative effort across institutions to build structured, harmonized representations of clinical interpretations of cancer genomic variants to advance the implementation of precision medicine. Our work has illustrated the diversity of variant interpretations available across resources, leading to inconsistency in interpretation of cancer variants. We have assembled a framework and recommendations for structuring and harmonizing such interpretations, from which the cancer genomics community can improve consensus interpretation for cancer patients. We have also developed and released open-source (MIT-licensed) and freely available aggregated knowledge resources (web application, data downloads and API) and associated analysis tools. Our working group and open-source software development environment are open to all and we welcome participation from anyone with an interest in learning about, utilizing, augmenting, improving or proposing new directions for this community-based project, for the benefit of cancer patients.

## Methods

### Collecting cancer variant interpretation knowledge

OncoKB, the CGI and JAX-CKB all contain complementary knowledge of variant oncogenicity. While valuable, knowledge of a variant’s potential role in driving tumorigenesis is structured differently than clinical interpretations of genomic variants, and is therefore outside of the scope of this manuscript. While omitted from the analyses presented in this paper, we do aggregate these annotations due to their potential utility in clinical research. ClinGen, ACMG, AMP, ASCO, VICC and CAP are working on developing guidelines to enable consistent and comprehensive assessment of oncogenicity of somatic variants. In the future, variant oncogenicity interpretations based on such guidelines can be incorporated into meta-knowledgebases and should help to improve the harmonization of related interpretations.

Exact code for collecting and harmonizing each of the VICC knowledgebases may be found at https://github.com/ohsu-comp-bio/g2p-aggregator. The cancer biomarker database from CGI was collected from the cgi_biomarkers_per_variant.tsv file from the biomarkers download at https://www.cancergenomeinterpreter.org/data/cgi_biomarkers_latest.zip. CIViC content was collected via the gene and variant API endpoints documented online at https://docs.civicdb.org/en/latest/api.html. JAX-CKB content of the publicly available 86 genes was collected from an unpublished API endpoint (collecting code at https://github.com/ohsu-comp-bio/g2p-aggregator/blob/v0.10/harvester/jax.py#L145-L147). MolecularMatch content was collected via an authorized API key for use in the aggregated knowledgebase (collecting code at https://github.com/ohsu-comp-bio/g2p-aggregator/blob/v0.10/harvester/molecularmatch.py). OncoKB content was collected via a combination of the levels, genes, variants and variants/lookup API endpoints documented at: http://oncokb.org/#/dataAccess. PMKB content was provided as a JSON file by the knowledgebase, which we are hosting at: https://s3-us-west-2.amazonaws.com/g2p-0.7/unprocessed-files/pmkb_interpretations.json.

### Harmonizing genes

Gene symbols were matched to the table of gene symbols from HGNC, hosted at the EBI^47^: ftp://ftp.ebi.ac.uk/pub/databases/genenames/new/json/non_alt_loci_set.json. This table was used to construct an ‘aliases’ table comprised of retired and alternate symbols for secondary lookup if the interpretation gene symbol was not found among the primary gene symbols from HGNC. If an alias used by a knowledgebase was shared between two genes, omitted by the knowledgebase or failed to match either the primary or alias table, the gene was omitted from the normalized gene field.

### Harmonizing variants

Variants collected from each knowledgebase were first evaluated for attributes specifying a precise genomic location, such as chromosome, start and end coordinates, variant allele and an identifiable reference sequence. Variant names were queried against the Catalog of Somatic Mutations in Cancer (COSMIC)^3^ v.81 to infer these attributes in knowledgebases that did not provide them. Custom rules were written to transform some types of variants without clear coordinates (for example, amplifications) into gene coordinates. All variants were then assembled into HGVS strings and submitted to the ClinGen Allele Registry (http://reg.clinicalgenome.org) to obtain distinct, cross-assembly allele identifiers, if available.

### Harmonizing diseases

Diseases were matched to the DO^48^, through lookup with the EBI OLS^47^, unless a preexisting ontology term for a different ontology existed (98.7% of interpretations map to DO). We downloaded the March 2018 release of the TopNode terms from https://github.com/DiseaseOntology/HumanDiseaseOntology/blob/master/src/ontology/subsets/TopNodes_DOcancerslim.json and mapped our interpretation diseases to this list, assigning each disease to its nearest TopNode ancestor (Supplementary Table 4 and Supplementary Note). We assigned remaining interpretation diseases to the nonspecific term of DOID:162 (cancer) if the disease was a descendant of this term, but not a descendant of one of the TopNode terms.

### Harmonizing drugs

Drug names were first queried against the biothings API^49^ for harmonization (documented at https://mychem.info/v1/api) and if not found were subsequently queried against the PubChem Compounds^29^, PubChem Substances and ChEMBL^30^ web services (see https://github.com/ohsu-comp-bio/g2p-aggregator/blob/v0.10/harvester/drug_normalizer.py for details).

### Harmonizing evidence level

Evidence levels were standardized to the AMP/ASCO/CAP guidelines as outlined in Table 1.

### Comprehensive evaluation of ERBB2 duplication

Public web portals for the six VICC knowledgebases were manually searched for interpretations for variants describing the alteration detailed in Fig. 2c. The MolecularMatch resource changed its data access policy after peer review of this manuscript, and is no longer accessible to the public. The web portals for the remaining resources are freely available online without registration at the following URLs:

- CGI: https://www.cancergenomeinterpreter.org/biomarkers
- CIViC: https://civicdb.org/search/variants/
- JAX-CKB: https://ckb.jax.org/geneVariant/find
- OncoKB: http://oncokb.org
- PMKB: https://pmkb.weill.cornell.edu

### Evaluating nonharmonized aggregate content

To evaluate the gains provided by our harmonization methods, we collected and minimally formatted interpretation elements from each knowledgebase without using any harmonization routines. We selected the set of unique elements for each resource and calculated the overlap across the union of those sets (Supplementary Table 3). We then repeated this procedure for harmonized elements and compared total element count and percentage overlap between harmonized and nonharmonized elements. Calculations for the specific fields of that table are provided in the Supplementary Note.

### Project GENIE

GENIE data were downloaded from the v.3.0.0 data release available at: https://www.synapse.org/#!Synapse:syn7222066/files/. Variants were extracted from ‘data_mutations_extended.txt’, and clinical data from ‘data_clinical_sample.txt’. Variants were filtered on predicted consequence of medium or high impact. This classification was based upon the VEP consequence table (http://useast.ensembl.org/info/genome/variation/prediction/predicted_data.html#consequences) and resulted in exclusion of variants classified as Silent, 3′Flank, 3′UTR, 5′Flank, 5′UTR, Intron or Splice_Region. Patients without any variants after filtering were included in all calculations. Oncotree cross-references were obtained from their API at http://oncotree.mskcc.org/api/tumorTypes (data version, oncotree_2018_05_01) and cross-references were then mapped to DO terms where they matched. In cases where one-to-many mappings occurred, manual review of those mappings was performed to select the most appropriate mapping.

### Variant intersection search

Variant coordinates were used to search genomic features via coordinate intersection. A complete intersection of query and target is considered a ‘positional match’, or a more specific ‘exact match’ if the alternate alleles also match. A ‘focal match’ is reported if the intersection fraction is less than complete, but over 10% overlapping (reciprocally). A ‘regional match’ is reported if there is any intersection, but the match is of no other type (Extended Data Fig. 4a).

### Disease TopNode search

Disease searching returns a distance of the number of ancestor or descendant TopNode terms between the queried disease and the matching target (see Supplementary Note for more on TopNode terms). Two diseases sharing a TopNode term (for example, DOID:3008, invasive ductal carcinoma, and its parent term DOID:3007, breast ductal carcinoma, are both members of DOID:1612, breast cancer) would have a distance of 0. However, if two diseases share a TopNode term but do not have a direct lineage, they are not a match. For example, DOID:0050938, ‘breast lobular carcinoma’, does not match to DOID:3007, ‘breast ductal carcinoma’, even though they share a TopNode term (DOID:1612, ‘breast cancer’), as they are sibling concepts and do not have an ancestor/descendant relationship (Extended Data Fig. 4b).

Enrichment testing for GENIE Oncotree diseases that map to DO TopNode was performed by comparing the count of a given disease term across the GENIE patients, and then splitting these counts into two groups: those diseases that mapped to DO in our analysis, and those that did not. This set of counts was ranked and compared by group using the Mann–Whitney *U*-test. The sets of counts (as well as the statistical test) may be found in cell 208 of the analysis notebook accompanying this study.

#### Gene intersection search

To assess cohort interpretability (Extended Data Fig. 6) when considering only matching a variant to a gene, we used the assigned gene symbols for each GENIE variant and compared them to interpretation gene symbols. Patients with at least one variant matching an interpretation gene symbol were considered a match. Matches were subsequently filtered by broad disease matching and by interpretation tier; no adjustment was made to the evidence level and tier to account for this imprecise aggregation strategy.

### ElasticSearch API and web front end

Collectors create ‘Association’ documents segmented by the source field. Documents are posted to an ElasticSearch v.6.0 instance provisioned by AWS elasticsearch service.

On top of ElasticSearch, we built web services using the Flask web framework. The search.cancervariants.org endpoint provides two simple REST-based web services: an association query service and a GA4GH beacon service. The association query service allows users to query for evidence using any combination of keywords, while the beacon service provisions G2P associations into the GA4GH beacon network (beacon-network.org) enabling retrieval of associations on the basis of genomic location. OpenAPI (swagger) documentation is provided to accelerate development and provide API integration scaffolding. Client applications can use the API to create higher level sets of queries driven by cohort allele sets (for example, MAF/VCF files) with varying genomic resolutions and disease/drug combinations. The API server and nginx proxy are described by Docker configurations and deployed colocated within a t2.micro instance.

The user interface is a customized Kibana dashboard that enhances Lucene-based full-text search of associations with interactive aggregation heat maps, tables and other components. The API documentation is available at: search.cancervariants.org/api/v1/ui/.

### Reporting Summary

Further information on research design is available in the Nature Research Reporting Summary linked to this article.

## Online content

Any methods, additional references, Nature Research reporting summaries, source data, extended data, supplementary information, acknowledgements, peer review information; details of author contributions and competing interests; and statements of data and code availability are available at 10.1038/s41588-020-0603-8.

## Supplementary information

Supplementary Information Supplementary Note and Equations

Reporting Summary

Supplementary Table Supplementary Tables 1–9

## Data Availability

Analyzed harmonized data from the aggregated knowledgebases are available for bulk download at https://s3-us-west-2.amazonaws.com/g2p-0.10/index.html. Data are made available according to the data sharing principles and data sharing agreement provided by the VICC (cancervariants.org/join). In accordance with these principles, all content is available for academic research. The CIViC, CGI Biomarkers and PMKB knowledgebases provide content with no restrictions on reuse; however, commercial use of content from other knowledgebases is restricted—see individual knowledgebases for current content licensing.

## Supplementary information

**Extended data** is available for this paper at 10.1038/s41588-020-0603-8.

## Code availability

The Python 3.6 interface package and Jupyter analysis notebook to generate these results are available online at http://git.io/vicckb, and are freely available for reuse under the MIT license. Code for the generation of Supplementary Table 8 and associated statistical tests is available online at https://github.com/ahwagner/vicckb/blob/master/supporting_scripts/VICCdisease_graphs_by_database.R. Docker containers and source code for the meta-knowledgebase are hosted online at https://github.com/ohsu-comp-bio/g2p-aggregator, and are freely available for reuse under the MIT license.

## References

[1] Huang L (2017). The cancer precision medicine knowledge base for structured clinical-grade mutations and interpretations. J. Am. Med. Inform. Assoc..

[2] Yeh P (2013). DNA-mutation inventory to refine and enhance cancer treatment (DIRECT): a catalog of clinically relevant cancer mutations to enable genome-directed anticancer therapy. Clin. Cancer Res..

[3] Forbes SA (2017). COSMIC: somatic cancer genetics at high-resolution. Nucleic Acids Res..

[4] Ainscough BJ (2016). DoCM: a database of curated mutations in cancer. Nat. Methods.

[5] Chakravarty, D. et al. OncoKB: a precision oncology knowledge base. *J. Clin. Oncol. Precis Oncol*. 10.1200/PO.17.00011 (2017). 10.1200/PO.17.00011 PMC5586540 28890946

[6] Landrum MJ (2016). ClinVar: public archive of interpretations of clinically relevant variants. Nucleic Acids Res..

[7] Whirl-Carrillo M (2012). Pharmacogenomics knowledge for personalized medicine. Clin. Pharmacol. Ther..

[8] Dienstmann R, Jang IS, Bot B, Friend S, Guinney J (2015). Database of genomic biomarkers for cancer drugs and clinical targetability in solid tumors. Cancer Discov..

[9] Patterson SE (2016). The clinical trial landscape in oncology and connectivity of somatic mutational profiles to targeted therapies. Hum. Genomics.

[10] Griffith M (2017). CIViC is a community knowledgebase for expert crowdsourcing the clinical interpretation of variants in cancer. Nat. Genet..

[11] Tamborero D (2018). Cancer Genome Interpreter annotates the biological and clinical relevance of tumor alterations. Genome Med..

[12] Damodaran S (2015). Cancer Driver Log (CanDL): catalog of potentially actionable cancer mutations. J. Mol. Diagn..

[13] Good BM, Ainscough BJ, McMichael JF, Su AI, Griffith OL (2014). Organizing knowledge to enable personalization of medicine in cancer. Genome Biol..

[14] Zehir A (2017). Mutational landscape of metastatic cancer revealed from prospective clinical sequencing of 10,000 patients. Nat. Med..

[15] Stockley TL (2016). Molecular profiling of advanced solid tumors and patient outcomes with genotype-matched clinical trials: the Princess Margaret IMPACT/COMPACT trial. Genome Med..

[16] Tsimberidou, A.-M. et al. Initiative for molecular profiling and advanced cancer therapy (IMPACT): an MD Anderson precision medicine study. *J. Clin. Oncol. Precis Oncol*. 10.1200/PO.17.00002 (2017). 10.1200/PO.17.00002 PMC5659750 29082359

[17] Barroilhet L, Matulonis U (2018). The NCI-MATCH trial and precision medicine in gynecologic cancers. Gynecol. Oncol..

[18] *Creating a Global Alliance to Enable Responsible Sharing of Genomic and Clinical Data* https://www.ga4gh.org/wp-content/uploads/White-Paper-June-3-final.pdf (Global Alliance for Genomics and Health, 2013).

[19] Lawler M (2015). All the world’s a stage: facilitating discovery science and improved cancer care through the global alliance for genomics and health. Cancer Discov..

[20] Siu LL (2016). Facilitating a culture of responsible and effective sharing of cancer genome data. Nat. Med..

[21] Clinical Cancer Genome Task Team of The Global Alliance for Genomics and Health. (2017). Sharing clinical and genomic data on cancer—the need for global solutions. N. Engl. J. Med..

[22] Li MM (2017). Standards and guidelines for the interpretation and reporting of sequence variants in cancer: a joint consensus recommendation of the Association for Molecular Pathology, American Society of Clinical Oncology, and College of American Pathologists. J. Mol. Diagn..

[23] Richards S (2015). Standards and guidelines for the interpretation of sequence variants: a joint consensus recommendation of the American College of Medical Genetics and Genomics and the Association for Molecular Pathology. Genet. Med..

[24] Patterson SE, Statz CM, Yin T, Mockus SM (2019). Utility of the JAX Clinical Knowledgebase in capture and assessment of complex genomic cancer data. NPJ Precis. Oncol..

[25] Povey S (2001). The HUGO Gene Nomenclature Committee (HGNC). Hum. Genet..

[26] Dunnen JT, Dalgleish R, Maglott DR, Hart RK (2016). HGVS recommendations for the description of sequence variants: 2016 update. Human..

[27] Sioutos N (2007). NCI Thesaurus: a semantic model integrating cancer-related clinical and molecular information. J. Biomed. Inform..

[28] Pawliczek P (2018). ClinGen Allele Registry links information about genetic variants. Hum. Mutat..

[29] Kim S (2016). PubChem substance and compound databases. Nucleic Acids Res..

[30] Davies M (2015). ChEMBL web services: streamlining access to drug discovery data and utilities. Nucleic Acids Res..

[31] Bose R (2013). Activating HER2 mutations in HER2 gene amplification negative breast cancer. Cancer Discov..

[32] Xu S (2016). Circulating tumor DNA identified by targeted sequencing in advanced-stage non-small cell lung cancer patients. Cancer Lett..

[33] Stephens P (2004). Lung cancer: intragenic ERBB2 kinase mutations in tumours. Nature.

[34] Kris MG (2015). Targeting HER2 aberrations as actionable drivers in lung cancers: phase II trial of the pan-HER tyrosine kinase inhibitor dacomitinib in patients with HER2-mutant or amplified tumors. Ann. Oncol..

[35] Gonzalvez F (2016). Abstract 2644: AP32788, a potent, selective inhibitor of EGFR and HER2 oncogenic mutants, including exon 20 insertions, in preclinical models. Cancer Res..

[36] Hyman DM (2017). Abstract CT001: Neratinib in HER2 or HER3 mutant solid tumors: SUMMIT, a global, multi-histology, open-label, phase 2 ‘basket’ study. Cancer Res..

[37] Siegel RL, Miller KD, Jemal A (2018). Cancer statistics, 2018. CA Cancer J. Clin..

[38] AACR Project GENIE Consortium. (2017). AACR Project GENIE: powering precision medicine through an international consortium. Cancer Discov..

[39] Mungall CJ (2017). The Monarch Initiative: an integrative data and analytic platform connecting phenotypes to genotypes across species. Nucleic Acids Res..

[40] Rehm HL (2015). ClinGen—the clinical genome resource. N. Engl. J. Med..

[41] *Genetic Database Recognition Decision Summary for ClinGen Expert Curated Human Variant Data* https://www.fda.gov/media/119313/download (US Food and Drug Administration, 2018).

[42] Ritter DI (2016). Somatic cancer variant curation and harmonization through consensus minimum variant level data. Genome Med..

[43] Brush, M. H. & Shefchek, K. & Haendel, M. SEPIO: a semantic model for the integration and analysis of scientific evidence. In *Proc. Joint ICBO-BioCreative 2016* Vol. 1747 (CEUR, 2016).

[44] Chibucos MC (2014). Standardized description of scientific evidence using the Evidence Ontology (ECO). Database.

[45] Mateo J (2018). A framework to rank genomic alterations as targets for cancer precision medicine: the ESMO Scale for Clinical Actionability of molecular Targets (ESCAT). Ann. Oncol..

[46] Lex A, Gehlenborg N, Strobelt H, Vuillemot R, Pfister H (2014). UpSet: visualization of intersecting sets. IEEE Trans. Vis. Comput. Graph..

[47] Park YM, Squizzato S, Buso N, Gur T, Lopez R (2017). The EBI search engine: EBI search as a service-making biological data accessible for all. Nucleic Acids Res..

[48] Kibbe WA (2015). Disease Ontology 2015 update: an expanded and updated database of human diseases for linking biomedical knowledge through disease data. Nucleic Acids Res..

[49] Xin J (2016). High-performance web services for querying gene and variant annotation. Genome Biol..

