# Supplementary material for: A harmonized meta-knowledgebase of clinical interpretations of somatic genomic variants in cancer
Source: Nat Genet. 2020 Apr 3;52(4):448–57. doi: 10.1038/s41588-020-0603-8 (PMC7127986; doi:10.1038/s41588-020-0603-8)
Supplement: Supplementary file 1 — Supplementary Note and Equations [file 41588_2020_603_MOESM1_ESM.pdf]

nature genetics

SUPPLEMENTARY INFORMATION

https://doi.org/10.1038/s41588-020-0603-8

In the format provided by the authors and unedited.

A harmonized meta-knowledgebase of clinical interpretations of somatic genomic variants in cancer

Alex H. Wagner[1], Brian Walsh[2], Georgia Mayfield[2], David Tamborero[3,4], Dmitriy Sonkin[5], Kilannin Krysiak[1], Jordi Deu-Pons[6,7], Ryan P. Duren[8], Jianjiong Gao[9], Julie McMurry[2], Sara Patterson[10], Catherine del Vecchio Fitz[11], Beth A. Pitel[12], Ozman U. Sezerman[13], Kyle Ellrott[2], Jeremy L. Warner[14], Damian T. Rieke[15], Tero Aittokallio[16,17], Ethan Cerami[11], Deborah I. Ritter[18,19], Lynn M. Schriml[20], Robert R. Freimuth[12], Melissa Haendel[2,21], Gordana Raca[22,23], Subha Madhavan[24], Michael Baudis[25], Jacques S. Beckmann[26], Rodrigo Dienstmann[27], Debyani Chakravarty[9], Xuan Shirley Li[8], Susan Mockus[10], Olivier Elemento[28], Nikolaus Schultz[9], Nuria Lopez-Bigas[3,6,7], Mark Lawler[29], Jeremy Goecks[2], Malachi Griffith[1]✉, Obi L. Griffith[1]✉, Adam A. Margolin[2] and Variant Interpretation for Cancer Consortium*

[1]Washington University School of Medicine, St. Louis, MO, USA. [2]Oregon Health and Science University, Portland, OR, USA. [3]Pompeu Fabra University, Barcelona, Spain. [4]Karolinska Institute, Solna, Sweden. [5]National Cancer Institute, Rockville, MD, USA. [6]Institute for Research in Biomedicine, Barcelona, Spain. [7]Catalan Institution for Research and Advanced Studies, Barcelona, Spain. [8]MolecularMatch, Houston, TX, USA. [9]Memorial Sloan Kettering Cancer Center, New York, NY, USA. [10]The Jackson Laboratory for Genomic Medicine, Farmington, CT, USA. [11]Dana-Farber Cancer Institute, Boston, MA, USA. [12]Mayo Clinic, Rochester, MN, USA. [13]Acibadem University, Istanbul, Turkey. [14]Vanderbilt University, Nashville, TN, USA. [15]Charité—Berlin University of Medicine, Berlin, Germany. [16]Institute for Molecular Medicine Finland, Helsinki, Finland. [17]University of Turku, Turku, Finland. [18]Baylor College of Medicine, Houston, TX, USA. [19]Texas Children's Hospital, Houston, TX, USA. [20]University of Maryland School of Medicine, Baltimore, MD, USA. [21]Linus Pauling Institute at Oregon State University, Corvallis, OR, USA. [22]Children's Hospital Los Angeles, Los Angeles, CA, USA. [23]Keck School of Medicine of USC, Los Angeles, CA, USA. [24]Georgetown University Medical Center, Washington, DC, USA. [25]University of Zurich, Zurich, Switzerland. [26]University of Lausanne, Lausanne, Switzerland. [27]Vall d'Hebron Institute of Oncology, Barcelona, Spain. [28]Weill Cornell Medicine, New York, NY, USA. [29]Queen's University Belfast, Belfast, UK. *A list of members and affiliations apears in the Supplementary Note. ✉e-mail: mgriffit@wustl.edu; obigriffith@wustl.edu

Supplementary Note

Differences in somatic and germline variant interpretation

When considered in discovery and translational research endeavors, it is important to determine if a particular variant observed in a gene of interest is *oncogenic* (the variant functionally enables or predisposes towards the development of cancer), as this annotation provides the foundation on which targeted cancer treatment research is based. In contrast, clinical applications are dominated by diagnostic, prognostic, or therapeutic interpretations which in part also depends on underlying variant oncogenicity.

The development of procedures for oncogenicity classification in somatic interpretation guidelines is an unmet need in the oncology domain. ClinGen, ACMG, AMP, ASCO, VICC and CAP are collaboratively developing such guidelines to enable consistent and comprehensive assessment of somatic variant oncogenicity.

Consensus and recommendations for the elements of an Interpretation

To provide readily searchable, standardized interpretations across knowledgebases, we evaluated the structure of cancer variant interpretations across the core dataset (**Figure 1**). Our first challenge was to develop a consensus for the minimum required data elements that constitute a cancer variant interpretation. These minimal elements include a gene identifier, variant name, cancer subtype (tumor type and organ), clinical implication (diagnostic, prognostic, therapeutic, or predisposing biomarker), provenance of supporting evidence (e.g., PubMed identifier), and curation source. In addition, we recommended ascribing a tiered level of support for the evidence contributing to the interpretation. Each VICC knowledgebase (**Supplementary Table 1**) provided cancer variant interpretations as structured data meeting these requirements.

Difference in curation strategy

The differing curation and data modeling strategies of these knowledgebases serve as contributing factors for their dramatically different variant content. CGI uses invited expert curators to build out its knowledgebase of therapeutic biomarkers. Similarly, JAX-CKB, MolecularMatch, OncoKB, and PMKB create interpretations from in-house expert panels, but the provenance of these interpretations are each credited to the entire panel instead of an individual curator. The CIViC knowledgebase provides an interface for anyone to curate cancer variant interpretations from the literature, and the curated content is then reviewed by expert editors. The need for only 1 (CGI) or 2 (CIViC) curators to generate interpretations may contribute to the greater breadth of variants in these resources compared to others. PMKB has far fewer variants than the others as a result of the way interpretations are modeled; many of the interpretations in PMKB apply to broad variant representations (described above as categorical variants) which may be used for multiple interpretations. In fact, there are more interpretations in PMKB than in OncoKB, but OncoKB has more specific variants associated with their interpretations. Additional differences exist between the focus of these knowledgebases that are not accounted for in our analysis. For instance, JAX-CKB and MolecularMatch each also aggregate clinical trial data, and CGI and OncoKB both maintain large "oncogenic" annotation lists, but none of these additional data are included in the analyses presented in this manuscript.

Grouping of disease terms to top-level disease concepts

The aggregated knowledge across the core dataset describes 357 distinct disease concepts from the Disease Ontology (DO)[47] across 12,497 interpretations (**Supplementary Table 4**). These diseases range from highly specific (e.g. *DOID:0080164 - myeloid and lymphoid neoplasms with eosinophilia and abnormalities of PDGFRA, PDGFRB, and FGFR1*) to generalized (e.g. *DOID:162 - cancer*). To compare the variant interpretations to disease type, we used the expert-curated "TopNodeCancerSlim" DO mapping[50] that describes 58 common, top-level disease terms (TopNode terms) across several major datasets, including The Cancer Genome Atlas (TCGA), International Cancer Genome Consortium (ICGC), and COSMIC.[3,51,52]

Mapping GENIE diseases to Disease Ontology
GENIE samples are annotated with a diverse array of Oncotree ontology (oncotree.mskcc.org) disease codes, with 81% (539 / 667) of Oncotree diseases represented in the dataset. Over 53% (286 / 539) of the Oncotree diseases from GENIE do not link to DO through cross-references, of which 34% (96 / 286) do not have any cross-references (**Supplementary Table 9**). This lack of cross-references among GENIE diseases is significantly higher than the 20% (133 / 667) of all Oncotree terms lacking cross-references (OR = 2.0, p = 1.1e-5; Fisher's exact test, two-sided), suggesting that terms used to describe individual patient cancers (e.g. *Well-Differentiated Neuroendocrine Tumor of the Rectum*) are less likely to map to other knowledgebases than high-level parent terms (e.g. *colorectal cancer*). Despite this, 65% of GENIE patients had a disease term map to DO, indicating that the common cancers among this cohort are more likely to be cross-referenced adequately for mapping. Further evaluation confirmed a significant enrichment of more frequently observed disease terms among the terms that mapped to DO, compared to those that did not (U = 31094.0, p = 4.8e-3; Mann-Whitney U test, two-sided; see **Online Methods**).

In addition to the above limitations, matching patient variants to diseases with our strategy is highly dependent upon ontology structure. For example, CGI exhibits a 54% reduction of exact matches due to the mapping of CGI terms to DO. To illustrate this problem, patients with DOID:3008, *invasive ductal carcinoma*, account for 11% of the unmatched diseases (or 6% of the overall reduction). 82% of these patients have a variant matching a CGI interpretation for DOID:3458, *breast adenocarcinoma*. This is a sibling term to *breast lobular carcinoma* and *breast ductal carcinoma*. As a result, GENIE *invasive ductal carcinoma* patients (*invasive ductal carcinoma* is the sole descendant of *breast ductal carcinoma*) do not match, as our match strategy requires ancestral relationships between concepts (see **Extended Data Figure 4** for details).

Improvements from harmonization
We observed large gains in overlapping terms between resources across variants, diseases, and drugs. Importantly, this harmonization allowed us to search relationships between patient and interpretation disease terms, improving precise matching between patient and interpretations of clinical significance. We noted that there was little need to harmonize gene identifiers, as each knowledgebase had independently selected HGNC gene symbols as a reference, enabling easy and direct comparison of genes. This underscores both the utility of standardized data, as well as the need for adoption of similar standards (such as those described in this work) to drive direct comparison of variant interpretations. In our analysis of the variants and diseases of the harmonized interpretations, we observed that frequent top-level cancer terms mirror cancers with high incidence and mortality. We also noted that a large percentage of these interpretations described a relatively small number of gene-disease relationships.

Future goals
Additionally, we are building inference tools to automatically identify the concepts users are querying in real time. We also will be expanding our effort to harmonize and present interpretations of various non-coding variants, structural variants beyond gene pairs, and aggregate markers like microsatellite instability status. A prioritized long-term goal is the development of standards and techniques for interpretations of combined germline and somatic variations. Similarly, we are building guidelines and methods to enable automated consensus recommendations. Finally, we are seeking out additional knowledgebases of clinical interpretations of variants to harmonize and share with the broader cancer genomics community, and building an API specification which they may use to incorporate their own interpretations.

50. Wu, T.-J. et al. Generating a focused view of disease ontology cancer terms for pan-cancer data integration and analysis. *Database* **2015**, bav032 (2015).
51. Cancer Genome Atlas Research Network. Comprehensive genomic characterization defines human glioblastoma genes and core pathways. *Nature* **455**, 1061–1068 (2008).
52. International Cancer Genome Consortium et al. International network of cancer genome projects. *Nature* **464**, 993–998 (2010).

Participating Knowledgebase Agreement

Dear VICC leaders,

I would like to take this opportunity to express my commitment to participating in the Variant Interpretation for Cancer Consortium (VICC). As you know, we are one of several institutions engaged in the challenge of curating knowledge to annotate cancer genome mutations associated with evidence of pathogenicity or linked to relevant treatment options. Specifically, we have developed the [INSTITUTION RESOURCE (INSTITUTION NAME)]. While this resource serves the specific needs of our own institution, we recognize that there is **clear value in sharing knowledge of cancer-variant-treatment associations.** Such sharing will increase confidence where interpretations overlap, fill gaps, reduce redundancy, and leverage disparate domain expertise. To this end, we support your plans to coordinate global efforts for curation and help develop a community resource for cross-knowledgebase queries under the auspices of the Global Alliance for Genomics and Health (GA4GH). As a VICC participant we agree with the following data sharing principles, developed through community discussion at GA4GH meetings and calls.

- We will commit to sharing at least a minimal set of data elements for cancer variant interpretations including: gene symbol, variant name, cancer subtype (tumor type and organ), clinical implication (drug sensitivity, drug resistance, adverse response, diagnostic, or prognostic), source (e.g., PubMed identifier) and curation group.

- We agree that to avoid patient data privacy concerns, the project will focus on only clinical interpretations of variants derived from published findings (literature, conference proceedings, and clinical trial records), not individual patient/variant-level observations. Thus, there should be no possibility of linking variants to individuals.

- We agree to share [SPECIFY: all OR a significant proportion] of our interpretations (with at least the minimal required data elements) accumulated by our ongoing curation efforts.

    - This content will be released under a permissive license (free and non-exclusive for at least research use).

    - Software developed as part of this data sharing initiative will be released in public repositories (e.g., github) with open source licenses.

    - Public APIs will be developed to facilitate access to our data for use by the VICC.

    - Wherever possible, data sharing will be facilitated by use of the existing GA4GH schemas, APIs and demonstration implementations.

    - Interpretations made available by our institution will also be made available as cross-knowledgebase bulk downloads.

I look forward to working together with the Variant Interpretation for Cancer Consortium to further our common goal of improving genomics-guided precision medicine for cancer patients.

Sincerely,

[INSTITUTION REPRESENTATIVE]

VICC Members
Tero Aittokallio[1,2,3,4]; Lawrence Babb[5]; Michael Baudis[6]; Jacques S Beckmann[7]; Anas Belouali[8]; Andrew Biankin[9]; Misha Bouzinier[10]; Steven E Brenner[11]; Alberto Cambrosio[12]; Jonah Campbell[12]; Ethan Cerami[13]; Debyani Chakravarty[14]; David Chang[9]; Brad Chapman[15]; Thomas Conway[16]; Christopher L Corless[17]; Melanie Courtot[18]; Robert Currie[19]; Catherine del Vecchio Fitz[13]; Jordi Deu-Pons[20,21]; Rodrigo Dienstmann[22]; Kenneth Doig[16]; Ryan P Duren[23]; Daniel Durkin[24]; Korneel Duyvesteyn[25]; Olivier Elemento[26]; Jonathan Ellis[27]; Kyle Elrott[17]; Kenneth W Eng[26]; Aidan Flynn[28,29]; Robert R Freimuth[30]; JianJiong Gao[14]; Martina Gasull[21]; Moritz Gerstung[18]; William B Glen[31]; Jeremy Goecks[17]; Santiago Gonzalez[21]; Sara Gosline[32]; Malachi Griffith[33]; Obi L Griffith[33]; Melissa Haendel[17,34]; Maximilian Haeussler[19]; David Heckerman[35]; Oliver Hofmann[36]; Peter Horak[37]; Sarah Hunt[18]; Ivan Jelas[38]; Vaidehi Jobanputra[39,40]; Rachel Karchin[41]; Ian King[42]; Liang K Koh[43]; Ana Krepischi[44]; Kilannin Krysiak[33]; Mario Lamping[38]; Melissa Landrum[45]; Mark Lawler[46]; Jennifer Lee[47]; Jonas Leichsenring[48]; Michele L Lenoue-Newton[49]; Paul Leo[27]; Huei S Leong[16]; Xuan S Li[23]; Xuelu Liu[13]; Nuria Lopez-Bigas[20,21,50]; Chris Love[16]; Ravi Madduri[51]; Subha Madhavan[8]; Sameer Malhotra[26]; Adam Margolin[17]; David L Masica[41]; Georgia Mayfield[17]; Matthew D McCoy[8]; Clay McLeod[52]; Christine Micheel[49]; Susan Mockus[24]; Victoria Muenzer[38]; Christopher J Mungall[53]; Rishi Nag[18]; Kevin Osborn[19]; Ravi Pandya[54]; Nicole Park[55]; Sara Patterson[24]; Michael Piechotta[56]; Beth A Pitel[30]; Gordana Raca[57,58]; Erin Ramos[59]; Shruti Rao[8]; Gunnar Ratsch[60,61]; Iker Reyes[21]; Damian T Rieke[38,62]; Deborah I Ritter[63,64]; Peter Rogan[65]; Jeffrey Rosenfeld[66]; Sameek Roychowdhury[67]; Gabe Rudy[68]; Gina Rueter[38]; Chris Sander[5,13]; Andrea Sboner[26]; Lynn M Schriml[69]; Nikolaus Schultz[14]; Ozman U Sezerman[70]; Sandra Siesing[48]; Lillian Siu[55]; Heidi J Sofia[59]; Dmitriy Sonkin[71]; Vipin Sreedharan[61]; Albrecht Stenzinger[48]; Andrew I Su[72]; David Tamborero[50,73]; Bin T Teh[43]; Nora C Toussaint[61]; Eliezer Van Allen[5,13,74,75]; Nicole A Vasilevsky[17]; Etienne Vignola-Gagne[12]; Ioannis Vlachos[74]; Andra Waagmeester[76]; Alex H Wagner[33]; Brian Walsh[17]; Jeremy L Warner[49]; Joachim Weischenfeldt[28]; Trish Whetzel[18]; Julia Wilson[77]; Chunlei Wu[72]; Andrew Yates[18]; Andrey Zapariy[78]

VICC Member Affiliations
[1]Institute for Molecular Medicine Finland, Helsinki, Finland; [2]Institute for Cancer Research, Oslo, Norway; [3]University of Oslo, Oslo, Norway; [4]University of Turku, Turku, Finland; [5]Broad Institute of MIT and Harvard, Cambridge, MA, USA; [6]University of Zurich, Zurich, Switzerland; [7]University of Lausanne, Lausanne, Switzerland; [8]Georgetown University Medical Center, Washington D.C., USA; [9]University of Glasgow, Glasgow, Scotland; [10]InterSystems Corporation, Cambridge, MA, USA; [11]UC Berkeley, Berkeley, CA, USA; [12]McGill University, Montreal, Québec, Canada; [13]Dana-Farber Cancer Institute, Boston, MA, USA; [14]Memorial Sloan Kettering Cancer Center, New York, NY, USA; [15]Harvard T.H. Chan School of Public Health, Boston, MA, USA; [16]Peter MacCallum Cancer Centre, Melbourne, Australia; [17]Oregon Health and Science University, Portland, OR, USA; [18]European Molecular Biology Laboratory - European Bioinformatics Institute, Cambridge, UK; [19]UC Santa Cruz, Santa Cruz, CA, USA; [20]Catalan Institution for Research and Advanced Studies, Barcelona, Spain; [21]Institute for Research in Biomedicine (IRB Barcelona), The Barcelona Institute of Science and Technology, Barcelona, Spain; [22]Vall d'Hebron Institute of Oncology, Barcelona, Spain; [23]MolecularMatch, Houston, TX, USA; [24]The Jackson Laboratory for Genomic Medicine, Farmington, CT, USA; [25]Hartwig Medical Foundation, Amsterdam, Netherlands; [26]Weill Cornell Medicine, New York, NY, USA; [27]Queensland University of Technology, Woolloongabba, Australia; [28]University of Copenhagen, Copenhagen, Denmark; [29]Rigshospitalet, Copenhagen, Denmark; [30]Mayo Clinic, Rochester, MN, USA; [31]Medical University of South Carolina, Charleston, SC, USA; [32]Sage Bionetworks, Seattle, WA, USA; [33]Washington University School of Medicine, St. Louis, MO, USA; [34]Linus Pauling Institute at Oregon State University, Corvallis, OR, USA; [35]Human Longevity Inc, San Diego, CA, USA; [36]University of Melbourne, Melbourne, Australia; [37]National Center for Tumor Diseases Heidelberg, Heidelberg, Germany; [38]Charité – Berlin University of Medicine, Berlin, Germany; [39]New York Genome Center, New York, NY, USA; [40]Columbia University Irving Medical Center, New York, NY, USA; [41]Johns Hopkins University, Baltimore, MD, USA; [42]University of Toronto, Toronto, Ontario, Canada; [43]National Cancer Centre Singapore, Singapore, Singapore; [44]National Institute of Science and

Technology in Oncogenomics, São Paulo, Brazil; [45]National Center for Biotechnology Information, Bethesda, MD, USA; [46]Queen's University Belfast, Belfast, UK; [47]Frederick National Laboratory for Cancer Research, Rockville, MD, USA; [48]Heidelberg University Hospital, Heidelberg, Germany; [49]Vanderbilt University, Nashville, TN, USA; [50]Pompeu Fabra University, Barcelona, Spain; [51]University of Chicago, Chicago, IL, USA; [52]St. Jude Children's Research Hospital, Memphis, TN, USA; [53]Lawrence Berkeley National Laboratory, Berkeley, CA, USA; [54]Microsoft, Redmond, WA, USA; [55]University Health Network, Toronto, Ontario, Canada; [56]Humboldt University of Berlin, Berlin, Germany; [57]Children's Hospital Los Angeles, Los Angeles, CA, USA; [58]Keck School of Medicine of USC, Los Angeles, CA, USA; [59]National Human Genome Research Institute, Bethesda, MD, USA; [60]Swiss Institute of Bioinformatics, Lausanne, Switzerland; [61]Swiss Federal Institute of Technology in Zurich, Zurich, Switzerland; [62]Berlin Institute of Health, Berlin, Germany; [63]Baylor College of Medicine, Houston, TX, USA; [64]Texas Children's Hospital, Houston, TX, USA; [65]University of Western Ontario, London, Ontario, Canada; [66]Rutgers University, New Brunswick, NJ, USA; [67]Ohio State University, Columbus, OH, USA; [68]Golden Helix, Bozeman, MT, USA; [69]University of Maryland School of Medicine, Baltimore, MD, USA; [70]Acibadem University, Istanbul, Turkey; [71]National Cancer Institute, Rockville, MD, USA; [72]Scripps Research, La Jolla, CA, USA; [73]Karolinska Institute, Stockholm, Sweden; [74]Harvard Medical School, Boston, MA, USA; [75]Brigham and Women's Hospital, Boston, MA, USA; [76]Micelio, Antwerp, Belgium; [77]Wellcome Sanger Institute, Hinxton, UK; [78]Dell EMC, Hopkinton, MA, USA

Supplementary Equations

Calculations for evaluating non-harmonized aggregate content

<u>Non-harmonized element counts:</u>
Given the set of interpretation elements:

$$E = \{genes,\ variants,\ diseases,\ drugs\}$$

and the set of knowledgebase resources:

$$R\ =\ \{cgi,\ civic,\ jax,\ molecularmatch,\ oncokb,\ pmkb\}$$

For each element $e \in E$, and each resource $r \in R$, we created a unique set of *non-harmonized* element values observed in that resource, $S_{e,\,r}$.

For **genes** ($S_{genes,\, r\, \in\, R}$), we used HGNC gene symbols, which were provided by each knowledgebase. Gene symbols were almost universally provided across interpretations, although some interpretations do not have associated genes.

For **variants** ($S_{variants,\, r\, \in\, R}$), we extracted the genomic coordinates (chromosome, start, stop) from each resource and created a unique set of those variants. JAX-CKB and OncoKB do not provide genomic coordinates for variants. When applicable, we split records by the appropriate delimiter to separate out multiple variants. For CGI, we also did minimal HGVS parsing for chr/start/stop when gDNA HGVS strings were provided.

For **diseases** ($S_{diseases,\, r\, \in\, R}$), we extracted the disease term from each knowledgebase and transformed it to lowercase text. PMKB represents diseases as a combination of tissue and tumor type, which we transformed to a compound string joined by a space (e.g., *Tissue: Breast* and *Type: Adenocarcinoma* became *Disease: breast adenocarcinoma*).

For **drugs** ($S_{drugs, r \in R}$), we extracted the drug term from each knowledgebase and transformed it to lowercase text. As many interpretations contain more than one drug, we identified the delimiting character for each resource where multiple drugs are represented as a single string and split the string on the delimiter (e.g., the single string "*dabrafenib* + *trametinib*" was treated as the two strings "*dabrafenib*" and "*trametinib*").

We did not perform this analysis for **evidence** levels, as there is no shared meaning behind unharmonized evidence levels across resources (**Table 1**).

The size of the set of unique values $\left|S_{e \in E, r \in R}\right|$ was recorded in **Supplementary Table 3**. For example, $\left|S_{genes, cgi}\right|$ (cell *B3* of **Supplementary Table S3**) represents the 183 unique gene symbols observed in the Cancer Genome Interpreter.

Harmonized element counts:

For each element $e \in E$, and each resource $r \in R$, we created a unique set of *harmonized* element values observed in that resource, $S'_{e, r}$. These values were determined via the element harmonization routines specified in the previous **Online methods** sections.

The sizes of the sets of unique values $\left|S'_{e \in E, r \in R}\right|$ were recorded in **Supplementary Table 3**. For example, $\left|S'_{genes, cgi}\right|$ (cell *C3* of **Supplementary Table 3**) represents the 182 unique gene symbols derived from the Cancer Genome Interpreter after harmonization.

Summary of gains from harmonization:

To measure the degree to which harmonization improved the consistency of observed terms across knowledgebases, we first calculated the total *Terms Evaluated*. For each element e, this consisted of calculating both the total *Terms Evaluated* from both non-harmonized (t_e) and harmonized (t'_e) valuesets:

$$t_e = \sum_{r \in R} \left|S_{e, r}\right| \quad \text{and} \quad t'_e = \sum_{r \in R} \left|S'_{e, r}\right|$$

For each element e we also recorded the total *Unique Terms* across the union of terms from all resources, by measuring the cardinality of both the non-harmonized (u_e) and harmonized (u'_e) valuesets:

$$u_e = \left|\bigcup_{r \in R} S_{e, r}\right| \quad \text{and} \quad u'_e = \left|\bigcup_{r \in R} S'_{e, r}\right|$$

Finally, for each element e we calculate the *Overlap* as the percentage reduction of the total Terms Evaluated to the total Unique Terms, for both the non-harmonized (o_e) and harmonized (o'_e) valuesets:

$$o_e = 1 - \frac{u_e}{t_e} \quad \text{and} \quad o'_e = 1 - \frac{u'_e}{t'_e}$$
