# Supplementary material for: A harmonized meta-knowledgebase of clinical interpretations of somatic genomic variants in cancer
Source: Nat Genet. 2020 Apr 3;52(4):448–57. doi: 10.1038/s41588-020-0603-8 (PMC7127986; doi:10.1038/s41588-020-0603-8)
Supplement: Supplementary file 2 — Reporting Summary [file 41588_2020_603_MOESM2_ESM.pdf]

nature research

Corresponding author(s): Malachi Griffith; Obi Griffith

Last updated by author(s): Jan 3, 2020

Reporting Summary

Nature Research wishes to improve the reproducibility of the work that we publish. This form provides structure for consistency and transparency in reporting. For further information on Nature Research policies, see Authors & Referees and the Editorial Policy Checklist.

Statistics

For all statistical analyses, confirm that the following items are present in the figure legend, table legend, main text, or Methods section.

n/a	Confirmed	
☐	☒	The exact sample size (*n*) for each experimental group/condition, given as a discrete number and unit of measurement
☐	☒	A statement on whether measurements were taken from distinct samples or whether the same sample was measured repeatedly
☐	☒	The statistical test(s) used AND whether they are one- or two-sided<br>*Only common tests should be described solely by name; describe more complex techniques in the Methods section.*
☒	☐	A description of all covariates tested
☒	☐	A description of any assumptions or corrections, such as tests of normality and adjustment for multiple comparisons
☒	☐	A full description of the statistical parameters including central tendency (e.g. means) or other basic estimates (e.g. regression coefficient) AND variation (e.g. standard deviation) or associated estimates of uncertainty (e.g. confidence intervals)
☐	☒	For null hypothesis testing, the test statistic (e.g. F, t, r) with confidence intervals, effect sizes, degrees of freedom and P value noted<br>*Give P values as exact values whenever suitable.*
☒	☐	For Bayesian analysis, information on the choice of priors and Markov chain Monte Carlo settings
☒	☐	For hierarchical and complex designs, identification of the appropriate level for tests and full reporting of outcomes
☒	☐	Estimates of effect sizes (e.g. Cohen's *d*, Pearson's *r*), indicating how they were calculated

Our web collection on statistics for biologists contains articles on many of the points above.

Software and code

Policy information about availability of computer code

Data collection

Exact code for harvesting and harmonizing each of the VICC knowledgebases may be found online at https://github.com/ohsu-comp-bio/g2p-aggregator. The cancer biomarker database from CGI was harvested from the cgi_biomarkers_per_variant.tsv file from the biomarkers download at https://www.cancergenomeinterpreter.org/data/cgi_biomarkers_latest.zip. CIViC content was harvested via the gene and variant API endpoints documented online at http://griffithlab.org/civic-api-docs/. JAX-CKB content of the publically available 86 genes were harvested from an unpublished API endpoint (harvester code online at https://github.com/ohsu-comp-bio/g2p-aggregator/blob/v0.10/harvester/jax.py#L145-L147). MolecularMatch content was harvested via an authorized API key for use in the aggregated knowledgebase (harvester code online at https://github.com/ohsu-comp-bio/g2p-aggregator/blob/v0.10/harvester/molecularmatch.py). OncoKB content was harvested via a combination of the levels, genes, variants, and variants/lookup API endpoints documented online at: http://oncokb.org/#/dataAccess. PMKB content was provided as a JSON file by the knowledgebase, which we are hosting online at: https://s3-us-west-2.amazonaws.com/g2p-0.7/unprocessed-files/pmkb_interpretations.json

Gene symbols were matched to the table of gene symbols from HGNC, hosted at the European Bioinformatics Institute (EBI): ftp://ftp.ebi.ac.uk/pub/databases/genenames/new/json/non_alt_loci_set.json. This table was used to construct an "Aliases" table comprised of retired and alternate symbols for secondary lookup if the interpretation gene symbol was not found among the primary gene symbols from HGNC. If an alias used by a knowledgebase was shared between two genes, omitted by the knowledgebase, or failed to match either the primary or alias table, the gene was omitted from the normalized gene field.

Variants harvested from each knowledgebase were first evaluated for attributes specifying a precise genomic location, such as chromosome, start and end coordinates, variant allele, and an identifiable reference sequence. Variant names were queried against the Catalog of Somatic Mutations in Cancer (COSMIC) v81 to infer these attributes in knowledgebases that did not provide them. Custom rules were written to transform some types of variants without clear coordinates (e.g. amplifications) into gene coordinates. All variants were then assembled into HGVS strings and submitted to the ClinGen Allele Registry (http://reg.clinicalgenome.org) to obtain distinct, cross-assembly allele identifiers, if available.

Diseases were matched to the Disease Ontology (DO), through lookup with the European Bioinformatics Institute (EBI) Ontology Lookup Service (OLS), unless a pre-existing ontology term for a different ontology existed (98.7% of interpretations map to DO). We downloaded

the March 2018 release of the TopNode terms from https://github.com/DiseaseOntology/HumanDiseaseOntology/blob/master/src/ontology/subsets/TopNodes_DOcancerslim.json and mapped our interpretation diseases to this list, assigning each disease to its nearest TopNode ancestor (Table S4). We assigned remaining interpretation diseases to the non-specific term of DOID:162 - Cancer if the disease was a descendent of this term, but not a descendant of one of the TopNode terms.

Drug names were first queried against the biothings API for harmonization (http://c.biothings.io/v1/query) and if not found were subsequently queried against the PubChem Compounds (https://pubchem.ncbi.nlm.nih.gov/rest/pug/compound/), PubChem Substances (https://pubchem.ncbi.nlm.nih.gov/rest/pug/substance/), and ChEMBL (https://www.ebi.ac.uk/chembl/api/data/chembl_id_lookup/search) web services.

GENIE data were downloaded from the 3.0.0 data release available online at: https://www.synapse.org/#!Synapse:syn7222066/files/. Variants were extracted from “data_mutations_extended.txt”, and clinical data from “data_clinical_sample.txt”. Variants were filtered on predicted consequence of medium or high impact. This classification was based upon the VEP consequence table (http://useast.ensembl.org/info/genome/variation/prediction/predicted_data.html#consequences) and resulted in exclusion of variants classified as Silent, 3’Flank, 3’UTR, 5’Flank, 5’UTR, Intron, or Splice_Region. Patients without any variants after filtering were included in all calculations. Oncotree xrefs were obtained from their API at http://oncotree.mskcc.org/api/tumorTypes (data version oncotree_2018_05_01), and xrefs were then mapped to DO terms where they matched. In cases where 1-to-many mappings occurred, manual review of those mappings was performed to select the most appropriate mapping.

Data analysis

Data analysis was central to this work, and described throughout the manuscript. All custom software used to generate and display these findings are publicly available online at github.com/ohsu-comp-bio/g2p-aggregator (website) and git.io/vicckb (python interface and analysis notebook).

For manuscripts utilizing custom algorithms or software that are central to the research but not yet described in published literature, software must be made available to editors/reviewers. We strongly encourage code deposition in a community repository (e.g. GitHub). See the Nature Research guidelines for submitting code & software for further information.

Data

Policy information about availability of data

All manuscripts must include a data availability statement. This statement should provide the following information, where applicable:

- Accession codes, unique identifiers, or web links for publicly available datasets
- A list of figures that have associated raw data
- A description of any restrictions on data availability

Analyzed harmonized data from the aggregated knowledgebases are available for bulk download online at https://s3-us-west-2.amazonaws.com/g2p-0.10/index.html. Data are made available according to the data sharing principles and data sharing agreement provided by the VICC (online at: cancervariants.org/join). In accordance with these principles, all content is available for academic research.

Field-specific reporting

Please select the one below that is the best fit for your research. If you are not sure, read the appropriate sections before making your selection.

☒ Life sciences ☐ Behavioural & social sciences ☐ Ecological, evolutionary & environmental sciences

For a reference copy of the document with all sections, see nature.com/documents/nr-reporting-summary-flat.pdf

Life sciences study design

All studies must disclose on these points even when the disclosure is negative.

Sample size

A set of 12,856 aggregate interpretations covering 3,437 unique variants in 415 genes, 357 diseases, and 791 drugs were aggregated and harmonized from six publicly available sources. GENIE data were downloaded from the 3.0.0 data release available online at: https://www.synapse.org/#!Synapse:syn7222066/files/. We collected 237,175 moderate or high impact variants from “data_mutations_extended.txt” and clinical data of all 38,207 patients from “data_clinical_sample.txt”.

Data exclusions

No collected data was excluded from the study.

Replication

All experiments can be reproduced by cloning the VICCkb repository (git.io/vicckb) through the shared Jupyter analysis workbook (analysis.ipynb).

Randomization

Content from each knowledgebase was generated independently and without coordination.

Blinding

The statistical analyses of this study were blind to the source knowledgebase, except for cases where the analysis was specifically describing characteristics of each individual knowledgebases (e.g. Figure S3, Table S8).

Reporting for specific materials, systems and methods

We require information from authors about some types of materials, experimental systems and methods used in many studies. Here, indicate whether each material, system or method listed is relevant to your study. If you are not sure if a list item applies to your research, read the appropriate section before selecting a response.

Materials & experimental systems

n/a	Involved in the study
☒	☐ Antibodies
☒	☐ Eukaryotic cell lines
☒	☐ Palaeontology
☒	☐ Animals and other organisms
☒	☐ Human research participants
☒	☐ Clinical data

Methods

n/a	Involved in the study
☒	☐ ChIP-seq
☒	☐ Flow cytometry
☒	☐ MRI-based neuroimaging
